# The Psychological and Physical Effects of Forests on Human Health: A Systematic Review of Systematic Reviews and Meta-Analyses

**DOI:** 10.3390/ijerph18041770

**Published:** 2021-02-11

**Authors:** Marita Stier-Jarmer, Veronika Throner, Michaela Kirschneck, Gisela Immich, Dieter Frisch, Angela Schuh

**Affiliations:** 1 Public Health and Health Services Research, Institute for Medical Information Processing, Biometry, and Epidemiology (IBE), Ludwig-Maximilians-Universität München, 81377 Munich, Germany; vthroner@ibe.med.uni-muenchen.de (V.T.); mkirschneck@ibe.med.uni-muenchen.de (M.K.); gimmich@ibe.med.uni-muenchen.de (G.I.); dfrisch@ibe.med.uni-muenchen.de (D.F.); angela.schuh@med.uni-muenchen.de (A.S.); 2 Pettenkofer School of Public Health, Institute for Medical Information Processing, Biometry and Epidemiology (IBE), Ludwig-Maximilians-Universität München, 81377 Munich, Germany

**Keywords:** forest therapy, forest bathing, Shinrin-Yoku, forest medicine, prevention, systematic reviews

## Abstract

Background: The aim of this systematic review of systematic reviews was to identify, summarise, and synthesise the available evidence of systematic reviews (SRs) and meta-analyses (MAs) on the preventative and therapeutic psychological and physical effects of forest-based interventions. **Methods:** Both bibliographic databases and grey literature sources were searched for SRs and MAs published until May 2020. Eight databases were searched for relevant articles: MEDLINE, Embase, Web of Science, Cochrane Library, PsycInfo, CiNii, EBSCO, and Scopus. Grey literature was sourced from Google Scholar and other web-based search tools. SRs and MAs that included randomised controlled (RCT), non-randomised controlled (NRCT), and non-controlled trials (NCT) on health-related effects of forest-based interventions were eligible if they had searched at least two databases. The methodological quality of eligible reviews was assessed by AMSTAR-2. **Results:** We evaluated 11 systematic reviews covering 131 different primary intervention studies, mostly from Asian countries, three of which included supplementary meta-analyses. The quality assessment resulted in moderate confidence in the results of two reviews, low confidence in six, and critically low confidence in three. The results of the eight moderate and low-rated reviews indicated that forest-based interventions are beneficial to the cardiovascular system, immune system, and mental health (in the areas of stress, depression, anxiety, and negative emotions). Evidence for the effectiveness of forest-based interventions on metabolic parameters in adults, the severity of atopic dermatitis in children and adolescents, and social skills and sociality in healthy primary school children was weak. **Discussion/Conclusions:** Evidence suggests beneficial therapeutic effects of forest-based interventions on hypertension, stress, and mental-health disorders, such as depression and anxiety. Changes in immunological and inflammatory parameters after forest therapy should be verified in bio-geographically native forests. In the future, more attention should be paid to careful planning, implementation, and reporting of primary studies and to systematic reviews on the effects of forest-based interventions.

## 1. Introduction

Today, an increasing number of people worldwide live in urban rather than in rural areas. This amounted to 55% of the world population in 2018, and the trend is rising [1]. While the world continues to urbanise, the value of natural environments, green spaces, and forests for the quality of life and well-being of urban populations is becoming more and more evident. Exposure to nature and green environments is increasingly recognised as an important resource for stress recovery and general health [2,3,4,5].

The Greater Tokyo Area is one of the world’s largest metropolitan regions with about 37 million inhabitants [1]. It is, therefore, not surprising that the idea of forest bathing originated in Japan. The Japanese Ministry of Agriculture, Forestry and Fisheries introduced forest bathing (Japanese: “Shinrin-Yoku”) in the early 1980s and funded a large research program to prove its medical and therapeutic effects [6]. The first centre for “forest therapy” was later opened, and Japanese universities are now offering a medical specialisation in “forest medicine”. In other Far Eastern countries, such as South Korea and China, forest bathing is also a recognised form of both therapy and disease prevention with a decades-long tradition. The concept of forest bathing has also arrived in the Western world. The Scandinavian countries were among the first European countries to implement projects on the healing effects of forests such as “Green Steps” in Sweden, “Power Trail at Ikaalinen Spa” in Finland, and “Nacadia Therapy Forest Garden” in Denmark [7]. Healthy people and those with pre-existing conditions participate in forest therapy programs of various kinds for preventive and therapeutic purposes in the both the USA and Europe. Forest bathing has become a global trend as a reaction to the current flood of stimuli and the hectic daily life in our modern society [8,9].

The methods applied in forest therapy and prevention programs vary considerably. A key component is the perception of the forest environment with all five senses (“five-sense experience”, including vision, smell, hearing, touch, and taste), which can be combined with meditation and walking or hiking in the forest, as well as various recreational activities and cognitive behavioural therapy [10]. In Germany, forest therapy and forest bathing have been successfully combined with classical naturopathic elements, such as water immersion (e.g., Kneipp therapy) and climatotherapy (climatic terrain cure, heliotherapy, fresh-air rest cure) to enhance the health benefits of forest therapy programs [11]. In 2017, the first European “cure and healing forest” was established in Northern Germany in the seaside resort of Heringsdorf on the Baltic Sea island of Usedom. The cure and healing forest can be used both by patients from rehabilitation clinics and by the general population to promote their own health and personal well-being, as well as for therapeutic interventions in respiratory, cardiovascular, orthopaedic, and psychosomatic diseases [12]. Scientists in German-speaking countries are also researching whether the native forest can be used for preventive or medical purposes [13]. In 2017, the Forestry Faculty at the University of Belgrade investigated a 30-hectare area at the Goč Mountain in Serbia and proclaimed the forest to be an appropriate “cure and healing forest”. This offers a new approach to health tourism in Serbia [14].

Forest therapy and its presumed preventive effects have recently received growing attention in the international scientific world. Many international studies have reported the health-promoting effects of exposure to the forest environment on both body and mind [9,11]. Primary studies, systematic reviews (SRs) and meta-analyses (MAs) have been conducted to determine the preventive and therapeutic effects of forest bathing, forest therapy, and forest medicine for various indications. Special attention has been paid to the benefits of forest therapy on mental health, as especially people living in urban areas are at increased risk of being exposed to stressful situations and developing chronic mental health disorders [15,16,17]. As a result of sedentary and/or hectic lifestyles, chronic stress combined with little physical activity plays an important role in the development of so-called diseases of civilisation [18], such as chronic cardiovascular and respiratory diseases, diabetes, skin diseases, and a weakened immune system [3,19]. Patients suffering from these types of diseases are among the target groups of interventional studies in the forest environment. Forest bathing is indicated not only for patients, but also for healthy individuals due to its mainly preventive character. Improving the quality of life and increasing well-being are particularly important goals.

The term “forest bathing” is derived from the Japanese “Shinrin-Yoku”, which literally means “diving into the atmosphere of the forest”. Other terms frequently used in international studies for interventions in forest environments are “forest therapy” and “forest medicine”. Forest interventions are sometimes also called “forest (healing) programs”. Schuh and Immich [11] proposed separate definitions for the two concepts “forest bathing” and “forest therapy”. “Forest bathing” should be used for preventive purposes in healthy people, while the term “forest therapy” should be used when interventions in the forest are intended to achieve therapeutic goals in people with existing conditions. The authors acknowledged that the two terms are not clearly separable in current usage. The term “forest bathing” (Shinrin-Yoku) is common in the literature. In this paper, we generally use “forest-based interventions” as a generic term for health interventions carried out in forests.

Nature therapy, nature-based rehabilitation, nature-based treatment programs, and similar terms are also frequently used in the scientific literature when studying nature-based therapeutic interventions [2,3,20]. As it is possible, but not mandatory, for nature-based therapeutic interventions to include forest areas, we have only considered papers in our systematic review on SRs and MAs that explicitly refer to forest-based interventions.

Our preliminary literature searches identified a large number of studies that could be deemed suitable for inclusion in a systematic review on SRs and MAs. The literature research by Meyer and Bürger-Arndt in 2014 [21] and the update which followed a few years later [22] are among the most comprehensive literature surveys in this regard. Their work, however, is a narrative review. Following a very comprehensive literature search, the authors provided a narrative summary of the study results published up to that point. Neither the effectiveness of the interventions nor the methodological quality of the reviews was examined in greater detail.

We aimed to gain a comprehensive, up-to-date overview and an in-depth understanding of which forest-based preventive and therapeutic interventions are effective for which health conditions. The objective of our review was to synthesise evidence from SRs and MAs that evaluated the effectiveness of forest-based interventions and to assess the quality of the systematic reviews based on such studies.

## 2. Materials and Methods

A study protocol of this systematic review was registered with the International Prospective Register of Systematic Reviews (PROSPERO), registration number CRD42020190649 [23].

### 2.1. Literature Search

One author (V.T.) conducted a systematic literature search of 8 bibliographic databases (MEDLINE, Embase, Web of Science, Cochrane Library, PsycInfo, CiNii, EBSCO, and Scopus) from inception to the end of May 2020. A second author (M.K.) used Google Scholar, PROSPERO, and the references of relevant reviews to search for related literature that may have been overlooked. All search terms are listed in Appendix A. There were no restrictions regarding the publication language.

### 2.2. Inclusion and Exclusion Criteria

**Figure 1:**
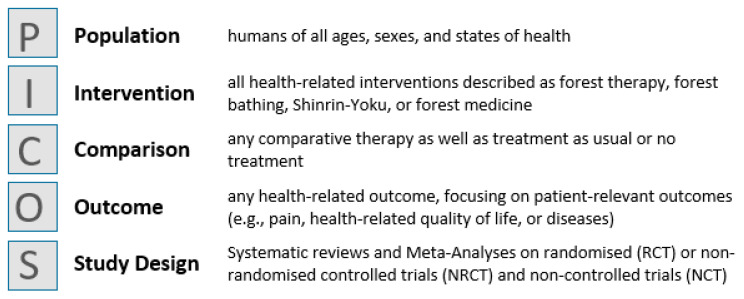
Inclusion and exclusion criteria based on PICOS (Population, Intervention, Comparator, Outcome, Study design) [24].

We generally aimed to identify all systematic reviews with or without meta-analyses that systematically searched for the effects of forest bathing (Shinrin-Yoku), forest therapy, or forest medicine of any kind, and summarised and reported them. The inclusion and exclusion criteria were based on the PICOS (Population, Intervention, Comparator, Outcome, Study design) elements [24] (Figure 1).

SRs and MAs on randomised (RCT) or non-randomised controlled trials (NRCT) and non-controlled trials (NCT) were eligible if they had used at least 2 databases for their literature search and at least 80% of the studies included had investigated forest-based interventions.

We excluded reviews (1) with a different study design than defined in inclusion criteria (e.g., narrative review), (2) where more than 20% of the primary studies contained interventions that were not carried out in forests (e.g., nature, park, field), and (3) that included theoretical studies or published opinions as their primary sources of evidence.

### 2.3. Deviation from the Study Protocol

There were two changes made to the study protocol: (1) We originally planned to exclude reviews where the study selection and/or data extraction had not been performed independently by two reviewers. This criterion was not applied in the review-selection process. (2) According to the protocol, only reviews containing exclusively forest-based studies were to be included. Instead, reviews were included if at least 80% of the primary studies included were forest-based.

### 2.4. Selection of Studies

After an initial selection based on the eligibility criteria and the removal of duplicates, we examined the titles and abstracts of the identified studies for their relevance. If the information in the title and abstract was insufficient, the full text was analysed (for information on excluded references in this step, see Appendix B). Abstract and full-text screening, selection process, data extraction, and quality assessment were carried out independently by 3 authors (V.T., M.K., M.S.J.). Each reference was reviewed by 2 of the 3 authors. Discrepancies were clarified through discussion with the involvement of the third author. In the quality assessment, the interrater reliability was calculated using Cohen’s kappa.

### 2.5. Quality Assessment

The methodological quality of included SRs and MAs was assessed using AMSTAR-2 (A Measurement Tool to Assess Systematic Reviews) explicitly critically appraising systematic reviews of randomised and non-randomised controlled clinical trials. The revised instrument (AMSTAR-2) comprises 10 of the original 11 existing dimensions and consists of a total of 16 questions with simpler answer options (“Yes”, “No”, “partial Yes”). A comprehensive guide is available for users [25].

On the basis of 7 so-called critical domains (items 2, 4, 7, 9, 11, 13, and 15) which can decisively influence the validity of a review and its conclusions, we made an assessment for each included review as to whether the confidence in the results of the review could be rated “high”, “moderate”, “low”, or even “critically low” [25,26]. The authors of the Guidance Document explicitly offer AMSTAR-2 users the option to deviate from the Guideline, if necessary. Appendix C gives a detailed description of the adjustments for the quality assessment of the current SRs and MAs.

### 2.6. Data Extraction

The following information on the SRs and MAs was extracted from the full texts of the relevant publications: reference, study objective/question, type of review, number of included primary studies, number and names of databases searched, search period, quality assessment tool(s) for included studies, results of the quality assessment (internal validity, quality of evidence), conflicts of interest, method of synthesis/analysis, and main results. We also extracted information on the studies included in the SRs and MAs: study design, publication period of the included primary studies, study populations, number of participants, interventions, countries/regions where the interventions were carried out, control/comparison groups and their interventions, duration of follow-up, indications, outcomes, and results.

## 3. Results

### 3.1. Search Results

**Figure 2:**
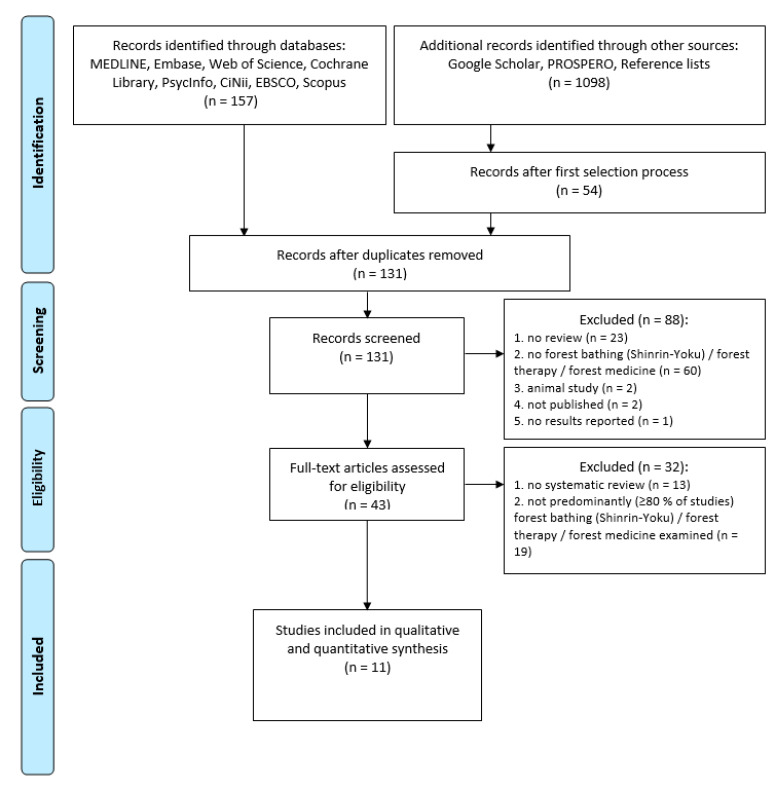
PRISMA (Preferred Reporting Items for Systematic Reviews and Meta-Analyses) flow chart for the selection of reviews [37].

The search identified 131 potentially relevant abstracts. After abstract screening, 88 records were excluded. The remaining 43 full-text articles were assessed for eligibility. Eleven SRs and MAs were finally considered eligible (Figure 2). All excluded articles are listed in Appendix B.

### 3.2. Characteristics of Included Studies

The characteristics of the included SRs and MAs, including key details of the covered primary studies, are presented in Table 1.

The SRs and MAs were published between 2016 and 2019. Four of these were conducted in Korea and one each in Japan, Australia, Indonesia, Italy, the USA, China, and the UK. Three included papers were systematic reviews with additional meta-analyses [27,28,29]. Most of the reviews examined the effects of forest-based interventions for specific indications, such as stress or depression [27,29,30,31,32,33,34]. Three studies are broader in scope and examined the health effects of forest-based interventions without focusing on specific conditions [19,35,36]. In five reviews, both study selection and data extraction were performed independently by two reviewers [19,27,32,33,34]. In four other reviews, either study selection [29,31] or data extraction [28,36] was performed by two reviewers, and in two reviews, neither was performed by two reviewers [30,35]. The authors of seven reviews stated that there were no conflicts of interest [19,28,30,32,34,35,36]. Four others did not provide conflict-of-interest statements [27,29,31,33]; one reported that no financial support was received for the preparation of the paper [29].

The 11 included SRs and MAs cover a total of 131 individual studies that examined the health effects of forest-based interventions. The studies were published between 1996 and 2019, with a particularly high proportion of publications in the years 2013 to 2015. Thirty-nine primary studies were RCTs, 55 NRCTs, and 32 NCTs. No information on the designs was available for a further five studies that were included exclusively in Putra’s systematic review [33].

More than three quarters (75.6%; *N* = 99) of individual studies were included in only 1 of the 11 reviews, 16.0% (*N* = 21) were included in two reviews, 6.1% (*N* = 8) in three reviews, one study was included in four, and two studies were included in five reviews.

The number of studies included in the reviews varied between 5 [31] and 28 [32,36]. There was a considerable variation in total sample sizes, ranging from 126 [33] to 2257 [29] participants. The study populations included healthy children, adolescents, and/or adults and people with health conditions and diseases. Most of the studies were conducted in Asia (South Korea, Japan, China, and Taiwan), and fewer in Europe (Poland, Spain, Germany, Iceland, Finland, Serbia, Great Britain, and Sweden) (Table 1).

### 3.3. Methodological Quality of Included Reviews

**Figure 3:**
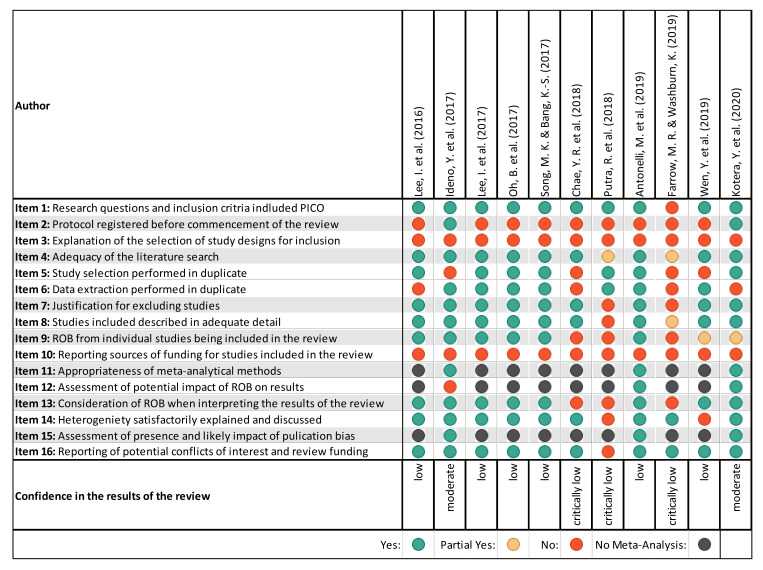
Quality assessment of included reviews based on AMSTAR-2. Critical domains are highlighted in grey. RoB: risk of bias.

Figure 3 provides a summary of the AMSTAR-2 results. The confidence levels in the results of the reviews were found to be moderate in two [28,29], low in six [19,27,31,32,34,36], and critically low in three [30,33,35]. Consensus estimates of interrater reliability showed good results (percentage agreement 84.7% and Cohens’ *k* = 0.77). None of the reviews met the criteria covered by AMSTAR-2 Item 3 (explanation of the selection of study designs for inclusion) and Item 10 (reporting sources of funding for studies included in the review). However, both domains are not considered critical in the sense of the AMSTAR-2 definitions. The failure to take these two points into account, together with the study selection and/or data extraction not being carried out independently by two authors, resulted in two reviews being rated with moderate rather than high confidence [28,29]. The main reason for judging six reviews to be of low quality according to the AMSTAR-2 guidelines was the lack of a study protocol, which should have been registered, published, or at least prepared prior to starting work on the review. A study protocol is also missing in the reviews that were assessed as critically low [30,33,35]. The latter left at least two other critical domains unconsidered: the application of a satisfactory technique for assessing the risk of bias (RoB) in individual studies included in the review and consideration of the RoB in the interpretation of the review results. Two of the reviews rated with critically low confidence did not report the reasons for the exclusion of studies.

### 3.4. Summary of Main Findings

Table 2, Table 3, Table 4, Table 5, Table 6, Table 7 and Table 8 summarise the main findings for the included SRs and MAs. The three reviews in which the quality assessment based on AMSTAR-2 (Figure 3) had shown critically-low confidence in the results were not considered in Table 3, Table 4, Table 5, Table 6, Table 7 and Table 8.

Most of the SRs and MAs included examined effects of forest-based interventions on specific indications or indication areas [27,29,30,31,32,33,34]. Three papers were broader in scope. They investigated the health effects of forest-based interventions without focusing on one specific indication or indication area [19,35,36]. The individual studies included in the reviews examined the effects of forest-based interventions mainly on the basis of surrogate endpoints (blood pressure, saliva/serum cortisol, immune and/or inflammation parameters, and other laboratory values). Patient-relevant endpoints are only found in the studies on skin diseases (atopic dermatitis) and mental health.

The evaluation of the evidence on the impact of forest-based interventions on different health outcomes summarised below is based not only on the study results, but also on criteria, such as the study designs used, the sample sizes, the distribution of age and/or gender in the study populations, the implementation of follow-up assessments, the study quality/risk of bias, and the number of included studies on the respective topic.

#### 3.4.1. Cardiovascular System

Three systematic reviews [19,28,36], one including a meta-analysis [28], reported on the effectiveness of forest-based interventions on the cardiovascular system in healthy people, as well as in adults with various previous diseases, such as hypertension. The higher-quality review by Ideno et al. [28] contains 20 studies on cardiovascular issues. Oh et al. [19] contains two studies, both of which are also included in Ideno et al. The review by Wen et al. contains eight studies on the topic, one of which is already included in Ideno et al. On the basis of the higher-quality review [26], we found that forest-based interventions showed positive effects on blood pressure and heart and pulse rates (Table 2 and Table 3). This was also seen in the lower-quality reviews. The results were confirmed by the meta-analysis by Ideno et al. The calculations using random-effects models showed statistically significant improvements with only slight heterogeneity among studies (SBP: mean difference (MD) = −3.15, 95% confidence interval (CI) [−4.12; −2.18], Heterogeneity (I^2^) = 1%; DBP: MD = −3.84, 95% CI [−5.27; −2.40], I^2^ = 24%; heart and pulse rate: MD = −1.75, 95% CI [−2.38; −1.13], I^2^ = 39%). Most of the individual studies on this topic were either non-randomised controlled trials with a cross-over design or randomised controlled trials. Limitations are often very small sample sizes (<30), unbalanced age, and/or gender distributions and the lack of follow-up (Table 1). There is also a high risk of selection bias and high risks for performance and detection bias due to the lack of blinding. As a result, there was evidence of short-term effects of forest-based interventions on cardiovascular parameters, which was confirmed by Ideno et al., whose review had the highest quality [28].

#### 3.4.2. Immune and/or Inflammatory Parameters

Three reviews of low and critically low quality [19,33,36] reported the effects of forest-based interventions on the immune system in healthy people, as well as in adults with various previous diseases, such as COPD or chronic heart failure. In most individual studies, an increase in natural killer (NK) or NKT cells and a decrease in cytokines and CRP were observed after the intervention. However, the results on changes in antioxidants and tumour necrosis factor α were inconsistent (Table 2 and Table 4). The critically low-quality review by Putra et al. [33] contains 10 individual studies on immune function, but these were described very inadequately and were not assessed for quality. This review could, therefore, not be used to meaningfully evaluate the evidence on the impact of forest-based interventions on the immune system. The work by Oh et al. [19] contains three individual studies, and that by Wen et al. [36] contains six studies on the topic. One of these studies is included in both reviews. All but one of the eight individual studies that investigated immunological parameters were randomised controlled trials. Methodological limitations included small sample sizes; skewed distributions in the age and/or gender of study participants; and, to a large extent, the lack of follow-up. (Table 1). Overall, on the basis of two low-quality reviews by Oh et al. and Wen et al., we found that there is evidence suggesting at least a short-term effect of forest therapy on some immunological and inflammation-related parameters.

#### 3.4.3. Metabolic Parameters

One review of low and one of critically low quality [35,36] examined the effects of forest-based interventions on metabolic parameters in healthy and hypertensive people. Due to its considerable methodological weaknesses (such as no quality assessment/risk of bias assessment for the included individual studies), the validity of the review of critically low quality by Chae et al. [35] on the effects of forest therapy in terms of changing metabolic parameters must be regarded as low. Wen et al. [36] reported on two studies that investigated the change in metabolic parameters after forest-based interventions and showed significant improvements in triglyceride and adiponectin levels (Table 2 and Table 5). Both were non-randomised controlled studies. The samples were small, the age and sex distributions were distorted, and there was no follow-up (Table 1). Against this background, the evidence for the effects of forest-based interventions on metabolic parameters is weak.

#### 3.4.4. Atopic Dermatitis

One lower-quality review [31] reported the effectiveness of forest-based interventions on the severity of atopic dermatitis in children and adolescents on the basis of the results of five individual studies. The severity of atopic dermatitis assessed by the SCORAD index improved significantly after intervention in four studies. No significance test was carried out in the fifth study (Table 2 and Table 6). Immunological blood tests showed divergent results. All trials were uncontrolled (pre–post design), had small samples, and had no follow-up (Table 1). The studies were also judged to be at high risk of detection bias due to the lack of blinding of the assessors. In summary, there is only weak evidence for the effect of forest therapy on the severity of atopic dermatitis in children and adolescents.

#### 3.4.5. Mental Health

##### Stress

Five lower-quality reviews [19,27,32,34,36], one including a meta-analysis [27], reported the effects of forest-based interventions on stress parameters in healthy people and in adults with various previous diseases. The summaries of the results showed that stress perception and stress hormones had significantly improved in the forest groups after intervention in nearly all individual studies (Table 2 and Table 7). Antonelli et al. [27] additionally performed a meta-analysis (fixed-effects model) on the basis of the results from eight selected RCTs. The reported effects on changes in saliva or serum cortisol levels as stress biomarkers were very small overall, while there was great heterogeneity among studies. Statistically significant effects were only observed in the subgroup *Forest Watching*. No effect was found for the overall group and the subgroup *Forest Walking* (overall: MD = –0.02, 95% CI [−0.05; 0.01], I^2^ = 81%; *Forest Walking*: MD = 0.03, 95% CI [−0.01; 0.08], I^2^ = 50%; *Forest Watching*: MD = −0.05, 95% CI [−0.08; −0.01], I^2^ = 84%). The asymmetry in the funnel plots also indicated a potential risk of publication bias. Antonelli et al. judged the risk of performance bias to be high, but for other types of bias to be unclear or low.

##### Anxiety and Depression

Four low-quality [19,32,34,36] and one moderate-quality reviews, the latter including meta-analyses [29], examined the effects of forest-based interventions on depressive symptoms and anxiety in healthy children and adults and in adults with various pre-existing conditions. Significant improvements in depressive symptoms were found in almost all the primary studies (Table 2 and Table 7). Similar results were found for anxiety. The meta-analyses (random effects model) by Kotera et al. [29] on depressive symptoms yielded small, but statistically significant, mean effect estimates with a high degree of heterogeneity among studies (RCTs: MD = −2.54, 95% CI [−3.56; −1.52], I^2^ = 87%; pre–post: MD = −1.04, 95% CI [−1.47; −0.60], I^2^ = 95%). All RCTs on depression had positive effects, i.e., depressive symptoms decreased more in the forest than in the city after the interventions. The meta-analysis of five RCTs on anxiety resulted in a large, but not significant, mean-effect estimator (MD = −8.81, 95% CI [−21.91; 3.57], I^2^ = 97%). In the meta-analysis of 16 uncontrolled studies on anxiety, the mean effect estimator achieved was small, but statistically significant, and there was also great heterogeneity among the studies (MD = −1.83, 95% CI [−3.07; −0.58], I^2^ = 98%).

##### Negative Emotions

The same five reviews [19,29,32,34,36] examined the effects of forest-based interventions on negative emotions, such as anger or aggression, in healthy children and adults and in adults with various pre-existing conditions. Participants showed a significant decrease in negative emotions after the forest-based interventions in most studies (Table 2 and Table 7). No changes could be found in some studies. The meta-analyses (random effects model) by Kotera et al. [29] on four RCTs and 12 pre-post studies on anger achieved small but statistically significant mean effect estimates with great heterogeneity among the studies (RCTs: MD = −1.63, 95% CI [−13.25; −0.01], I^2^ = 88%; pre–post: MD = −0.81, 95% CI [−1.17; −0.45], I^2^ = 93%). In summary, forest therapy seems to have positive effects on emotional states, such as anger, aggression, impulsiveness, and rage in children, and in healthy and pre-diseased adults.

##### Quality of Life/Well-Being

Three lower-quality reviews [32,34,36] reported on the impact of forest-based interventions on the health-related quality of life in healthy children and adults and in adults with severe depression and in psychiatric patients. Significant changes in health-related quality of life after forest-based interventions in adults could be shown (Table 2 and Table 7). One primary study included in the review by Song and Bang could not detect any change in the well-being of healthy children after an eight-week forest-based intervention [34].

##### Mental Health—Conclusion

Six reviews [19,27,29,32,34,36], two including meta-analyses [27,29], reported the impact of forest-based interventions on various aspects of mental health, including stress, anxiety, depression, negative emotions, and quality of life/well-being (Table 2 and Table 7). Confidence in the results of the reviews according to AMSTAR-2 was rated as moderate in one review [29] and as low in the other five reviews (Figure 3). On the basis of the better-quality SRs and MAs, we found that the meta-analyses on depression revealed small but statistically significant mean effect estimates with great heterogeneity among studies [29]. In the lower-quality SRs and MAs, the changes in saliva or serum cortisol levels as stress biomarkers reported in the meta-analysis were very small overall, and there was also great heterogeneity among the studies [27]. Many of the primary studies had a non-randomised or uncontrolled design, the sample sizes were sometimes small, the age and gender distribution were not always balanced, and there was no study with a follow-up. The risk of bias that could have influenced the results of the primary studies was assessed differently by the authors of the six reviews. The risk for selection, performance, and detection bias was often considered high, while attrition and reporting bias seemed to be of little relevance. In summary, there is some evidence for the effectiveness of forest therapy on mental health. Indications of beneficial effects are mainly found in the areas of stress, depression, anxiety, and negative emotions.

#### 3.4.6. Sociality

One lower-quality review [32] reported the impact of forest-based interventions on social skills and sociality in healthy primary-school children based on the results of 15 individual studies (two additional studies did not include forest-based interventions). Significant improvements were found in all outcomes after the respective intervention and compared to the control groups (Table 2 and Table 8). Significant changes could be observed only in some parts of the scales used, i.e., in the ability to adapt to school. The interventions in the primary studies included in this review were mostly forest-based programs that went beyond the usual forest-therapy walks, meditation and stays in the forest and included additional elements, such as psychotherapy, exercise therapy or water therapy (Table 1). The psychotherapeutic elements were likely to play a substantial role in the changes achieved. None of the primary studies had a randomised design, and there was a high risk of biased results in most of the trials, e.g., because evaluators were not blinded and major confounding variables had not been taken into account. Despite many positive study results, the evidence on the impact of forest-based interventions on social skills and sociality in healthy primary-school children is limited due to shortcomings in study implementation and because of the specific interventions.

## 4. Discussion

Our review summarises the evidence from systematic reviews and meta-analyses available for the health effects of forest-based interventions, including a comprehensive evaluation of the methodological quality of the included studies. We identified and synthesised a total of 11 publications with 131 included primary studies, mostly from Asian countries.

There is some overlap among the identified reviews, both at the primary study level (i.e., if the same primary study was included in more than one review) and at the review level (more than one identified review on the same topic). In a review on reviews, this can lead to a misinterpretation of results and overestimation of evidence [38]. Of the 131 primary studies, 32 (24.4%) were included in more than one review, of which 21 (16.0%) were included in exactly two reviews. However, the included reviews/meta-analyses often addressed different research questions and thus used different outcomes of the primary studies considered. For example, Antonelli et al. [27] summarised the effects of forest-based interventions on cortisol levels, whereas Ideno et al. [28] were interested in the effects of the forest environment on blood pressure. The primary studies included in both reviews overlapped. However, since the two reviews considered different outcomes from the primary studies, their overlap did not affect the interpretation of results in our review. In summary, we consider the risk of overestimating the evidence due to overlap to be low.

### 4.1. Key Findings from the Review—Summary of Evidence

The systematic reviews that summarised the effects of forest-based interventions on the cardiovascular system consistently reported positive effects both for prevention in healthy people and for the treatment of adults with various pre-existing conditions [19,28,36]. Forest-based interventions also showed positive short-term effects on immunological and inflammation-related parameters [19,36]. The possible improvement of metabolic parameters after forest-based interventions remains unclear, as this outcome has been investigated in too few primary studies [35,36]. One review synthesised the impact of forest-based interventions on the severity of atopic dermatitis in children and adolescents. Although there were significant improvements in the clinical picture, the evidence of the effect was weak due to partially divergent results and serious methodological shortcomings, including the study designs [31]. Regarding mental health, several reviews showed positive effects on stress, depression, and anxiety, as well as on negative emotions in healthy people and adults with various pre-existing conditions after forest-based interventions [19,27,29,32,34,36]. Some reviews also reported significant improvements in health-related quality of life among adults [32,34,36]. A further review points, albeit with limited evidence, to the benefits of forest therapy programs on social skills and sociality in healthy primary school children [34].

### 4.2. Latest Work on the Topic

Several primary studies and four reviews [39,40,41,42] on the topic—conducted in China, Italy, the USA, and Malaysia—were newly published during the few months in which we carried out our systematic review of SRs and MAs. According to PROSPERO, a meta-analysis of randomised controlled trials on the effects of forest-based interventions on mental health is also currently being conducted in Korea [43], and a research group in the UK registered an umbrella review on the health benefits of forest therapy in 2019 [44]. However, the topics of these newly published reviews remained more or less the same as those covered in the previous reviews—the effects of forest bathing in hypertensive patients (main findings: forest bathing interventions reduced blood pressure, lowered the pulse rate, increased the power of heart rate variability (HRV), improved cardiac-pulmonary parameters, improved metabolic function, induced a positive mood, reduced anxiety levels, and improved quality of life) [39], the health benefits of forests in terms of stress reduction and relaxation (main finding: positive association between forest exposure and mental well-being, suggesting forests as being effective in lowering stress levels) [40], and physiological and psychosocial effects of forest therapy (main finding: forest therapy plays an important role in preventive medicine and stress management for all age groups) [42]. Only Hansen and Jones addressed a topic which, to our knowledge, has not been dealt with in previous reviews, or at least not in a focused manner. In their review, they investigated the relationship between Shinrin-Yoku and spirituality (main finding: nature may have a positive effect on human spirituality and, therefore, can enrich individuals’ well-being) [41].

### 4.3. Mechanisms of Action

Forest bathing originated in Japan in the 1980s, and the practice is already much more widespread there and in other Asian countries than in Europe. The question often arises as to whether the health mechanisms in European forests are equivalent or at least comparable to those in Asian forests [9,29,45,46]. Forests with a closed canopy are comparable because they provide a special interior forest climate with reduced air temperature, high air purity and humidity, and special light conditions. These climatic factors are beneficial to health and relieve the respiratory tract and the thermoregulation system [11]. Another important factor is the peace and quiet in forests, which is essential for mental recovery in a time of acoustic stimulus overload in urban settings. It is assumed that the effect of biogenic volatile organic compounds (BVOC) emitted by trees and plants, such as phytoncides (also known as terpenes in German), has an influence on human health in terms of their anti-inflammatory, antioxidant, or neuroprotective activities [47,48,49]. This has not yet been conclusively clarified scientifically, but the authors of a recent state-of-the-art review confirmed that inhaling forest VOCs can result in useful antioxidant and anti-inflammatory effects on the airways. Other possible benefits mentioned include promoting brain function by reducing mental fatigue, inducing relaxation, and improving cognitive performance and mood. The authors emphasised that tree composition can significantly influence the concentration of certain VOCs in forest air [50]. The scientific literature on forest-based interventions too rarely describes the structure of the forests in which the interventions were carried out. The different types of forests are rarely compared regarding their health effects, making it almost impossible to compare forests both within a country and across continents.

### 4.4. Strengths and Limitations

#### 4.4.1. Strengths

The present systematic review on SRs and MAs across the globe provides the most comprehensive summary of the currently available evidence on forest-based preventative and therapeutic interventions. The systematic search in the major medical literature databases was carried out without language restrictions. This was necessary, as many primary studies and systematic reviews on the impact of forest-based interventions were carried out in Asia and are often published in the local language. A further strength of our review was the stringent quality assessment using a specific tool for the evaluation of systematic reviews with and without meta-analyses.

#### 4.4.2. Limitations at the Primary Study Level

The authors of the included reviews considered many of the included primary studies to be of medium or poor quality or at (relatively) high risk of bias (Table 1). The most common concern was the lack of blinding of participants, therapists, and/or assessors, which can contribute to both performance and detection bias. Many of the included primary studies were also characterised by a lack of reporting quality (e.g., insufficiently described target groups and randomisation; confounding variables were not reported; results were insufficiently reported; dropouts, randomisation, and/or blinding were not reported), deficiencies in study design (e.g., small study populations; skewed distribution of characteristics such as age or gender in the groups; unrepresentative results; no or inadequate control groups; no follow-up; important confounding parameters were not recorded; study dropouts were not considered; use of non-validated instruments), and deficiencies in the conduct of the study.

#### 4.4.3. Limitations at the Review Level

Most of the reviews included in the AMSTAR-2 evaluation were assessed as having low or critically low confidence in the findings (Figure 3). Only studies from a single country—usually South Korea—were included in several reviews [31,33,34,35]. The literature search was mostly limited to publications written in English or additionally in the respective national language. Many of the reviews lacked an a priori prepared study protocol; a quality assessment of the included primary studies; and an independently conducted study selection, data extraction, and quality assessment by at least two reviewers.

#### 4.4.4. Limitations at the Overview Level

An overview of systematic reviews itself has methodologic limitations, such as the potential loss of information due to repeated syntheses. Overviews of reviews depend on the quality of the included systematic reviews and these depend on the quality of the included primary studies. Ideally, only high-quality studies should be included in an overview of reviews, but this is not realistically feasible for many issues.

We were unable to conduct literature searches in Korean, Japanese, or Chinese databases. Thus, we may have missed a systematic review. However, since we used seven major international medical databases and searched for systematic reviews (not for single studies), we believe that we identified most for this comprehensive overview. We also conducted a recent update and identified currently ongoing reviews (see Section 4.2). Very recent findings may have been missed because recently published single studies could not have been captured by the reviews included.

We automatically translated the three reviews written in Korean using Google Translate. Since both the abstracts and the tables in these publications were available in English, we consider this procedure to be legitimate and sufficiently valid. Nevertheless, it cannot be ruled out that some information was not recognised or was misinterpreted.

### 4.5. Implications for Practice and Future Research

#### 4.5.1. Interventions

The forest-based interventions reported in the included SRs and MAs covered a broad and heterogeneous range of activities. These range from individual forest-based interventions, such as “experiencing the forest with all five senses”, to forest therapy with several activities in the forest, such as walking, sitting, and observing, to complex multimodal forest healing programs. The latter were also combined with sports activities and/or therapeutic elements, such as psychotherapy, exercise therapy, water therapy, or nutritional therapy in some studies.

There are no internationally agreed definitions of forest bathing, forest therapy, and other forest-based interventions and programs. Both the goals and the contents of forest therapy differ in the different countries. While in Japan, forest bathing is supposed to help stressed-out people find peace of mind, the focus in South Korea is on general health promotion and the enhancement of the common welfare through various forest programs for all population groups [51]. In America, a connection to nature is to be established through immersion in the forest environment [52].

In Japan, where Shinrin-Yoku has a decades-long tradition, it is defined as forest bathing in specially selected forests with the aim of maintaining physical vitality and mental health, as well as preventing disease. The focus is especially on stress reduction and strengthening the immune system. The Forest Therapy Society of Japan attempts to establish an international standard for practice through information events and training. The definition and potential elements of forest therapy remain imprecise and very broad. The forest should be experienced with all five senses during the forest bath/forest therapy. Activities such as hiking/walking, but also mindfulness exercises for the perception of the environment, can be practiced. Measures such as nutritional therapy can complement these activities [53].

In summary, the interventions carried out under the labels “forest bathing” or “forest therapy” are diverse and often difficult to compare. Stays in the forest, combined with mindfulness exercises and perception exercises with all senses, constitute the core elements of forest bathing/forest therapy. Additional activities, such as Nordic walking, hiking, and swimming; psychotherapeutic measures; and nutritional therapy, which together often resemble a multimodal approach from the naturopathic complex treatment, are obviously not excluded. Future studies need to define and precisely describe these interventions, especially when implementing such multimodal forest-based programs.

#### 4.5.2. Selection of Control Groups for the Investigation of the Effects of Forest-Based Interventions

Most primary studies with a control group examined the effects of forest-based interventions in comparison to the same interventions in an urban environment. These included walks and meditation.

Some reviews also include primary studies with multimodal forest-based interventions that go beyond the usual walks, meditation, and stays in the forest, and do not differ significantly from a traditional, holistic approach from naturopathy with exercise, nutrition, relaxation, and stress management. The effects achieved by such interventions, often referred to as “forest healing programs”, were usually compared to control groups without any specific intervention. Controls merely followed their “daily routine” [32,34]. These studies examined the health effects of complex forest-based interventions without specifically addressing the contribution of the forest environment to the effects achieved. It remains unclear as to whether the same complex interventions in an environment outside the forest would have led to the same results. The control group design used here can, therefore, be described as inadequate for the investigation of the forest effect. It is similar with a study included in the review by Wen et al. [36] in which participants with neck pain received forest therapy. The intervention consisted of forest bathing in both the control group and the intervention group. The intervention group additionally performed stretching and strengthening exercises [54]. This, too, is an example of an inadequate control group, since this study design was used to investigate the effects of stretching and strengthening exercises rather than forest therapy.

These examples show that special attention should be paid to a suitable control group when choosing a study design to demonstrate the effects of forest bathing/forest therapy.

#### 4.5.3. Recommendations for Practice and Future Research

The therapeutic effects of forest-based interventions on hypertension, stress, and mental health disorders, such as depression and anxiety, have been demonstrated. Forest therapy could also have a positive effect on the severity of atopic dermatitis in children. However, these effects reported in a Korean review [31] need to be confirmed in better-designed, controlled intervention studies. The effects on immunological and inflammation-related parameters should be verified by studies in appropriate native forests.

Forest characteristics, such as terpene concentration, microbial diversity, biodiversity, noise or quiet (psychoacoustics), light conditions, forest composition, and climatic factors, should be included in studies. Many questions remain unanswered in this context. For example, what kind of forest is needed for what objective? Do deciduous forests have a different effect than coniferous forests (an initial pilot study by Karim et al. [13] suggests differences)? How long does the stay in the forest have to last to have medium-to-long-term effects in addition to the acute effects? Which groups of people or patients benefit most from which intervention? Vulnerable groups, such as the elderly and/or people with disabilities, should also be considered.

Target groups for preventative forest-based interventions are people under stress and individuals with mental and emotional problems, particularly (a) elderly people (to encourage participation and prevent falls), (b) individuals living alone, and (c) patients in the rehabilitation phase at health resorts. To achieve sustainable effects, forest bathing should be practiced several times a week.

## 5. Conclusions

The present systematic international review of SRs and MAs provides the most comprehensive summary of the currently available evidence on forest-based preventive and therapeutic interventions.

Most of the systematic reviews did not reach an acceptable quality level after a stringent assessment of their methods. The study designs, control groups, and study populations of the included individual studies were often inadequate for the research question under investigation, which considerably limits the contribution of the relevant studies to the evidence in the research field.

The results of the better reviews showed that forest bathing may be beneficial for (1) the cardiovascular system by lowering blood pressure and reducing the heart rate/pulse rate in healthy adults and in people with hypertension and (2) mental health by reducing stress and symptoms of depression and anxiety and decreasing anger in healthy adults and in people with various health conditions. Forest therapy may also be beneficial for atopic dermatitis in children. These effects need to be confirmed in well-designed, controlled intervention studies. No considerable effects of forest-based interventions were seen for changes in antioxidants and tumour necrosis factor α, sociality, and metabolic parameters.

In summary, the results suggest that forest-based interventions have a positive impact on the cardiovascular system; some immunological and/or inflammatory parameters; and mental health in the areas of stress, depression, anxiety, and negative emotions. Positive effects were seen in healthy children and adults, as well as in adults with various pre-existing conditions. The evidence for the effectiveness of forest-based interventions on metabolic parameters in adults, on the severity of atopic dermatitis in children/adolescents, and on social skills and sociality in healthy primary school children was weak. Since there is no uniform use of the terms forest bathing (Shinrin-Yoku), forest therapy, and forest medicine, future studies need to define and precisely describe their interventions.

In the future, intervention studies with well-founded designs regarding the type of study, control group, type and duration of the intervention, study population, and description of the forest environment are required to improve our knowledge of forest-based interventions for health promotion and therapeutic purposes. The sustainability of the effects needs to be examined by sufficient post-intervention, follow-up assessments. When conducting systematic reviews on the topic, additional attention should be paid to methodologically careful study planning, implementation, and reporting.

## Figures and Tables

**Table 1:** Characteristics of included reviews and meta-analyses including key details of the covered primary studies.

First Author (Date); Country	Search Details	Objective;	Assessment of Risk of Bias and/or Study Quality:	Number (Type) of Studies Included;	Study Population;	Interventions: Intervention Group (IG) and Control Group (CG);
		Meta-Analysis: Yes/No			Total Number (Range)	
				Country of Study Implementation		Intervention Duration
Lee (2016);	6 DBs, from inception to Nov. 2015;	Effects of forest programs on atopic dermatitis;	RoBANS	*N* = 5 (5 NCT);	Children, adolescents with atopic dermatitis;	IG: forest experience, forest trip; forest camp; physical therapy; forest-camp swimming; forest activities, disease education
			SB: confounder and selection of participants—high, comparability—predominantly low			
South Korea [31]	GL: unpublished dissertations considered					
			PB: high			
					184 (12–64)	
						CG: n. a.;
		No	DB: high	Korea		Durations: 1–3 days (3–4 nights)
	LR: n. a.					
			AB: predominantly low			
			RB: predominantly low			
Ideno (2017);	4 DBs, from inception to May 2016;	Effects of the forest environment on blood pressure;	RoB	*N* = 20 (2 RCT, 15 NRCT cross-over, 3 NRCT);	Healthy adults, adults with hypertension;	IG: walking in/sitting in and viewing forest area
			SB: high (with 50%)			CG: walking in/sitting in and viewing in non-forest environment, such as city areas, sitting in a room, measuring blood pressure in daily life;
	GL: was searched		PB: high			
Japan			DB: high	Japan (*n* = 17)	732 (6–268)	
[28]	LR: English and Japanese language only		AB: low	Korea (*n* = 2)		
		Yes	RB: low	China (*n* = 1)		
						Durations: 15 min to 2 h; 1 × 3 days, 1 × 7 days
Lee (2017); South Korea [32]	7 DBs, from inception to July 2016; GL: n. a. LR: English and Korean language only	Effects of forest therapy on depressive symptoms; No	SIGN checklist 18 studies low and 10 acceptable quality	*N* = 28 (17 RCT, 11 NRCT); Korea (*n* = 17) Japan (*n* = 7) China (*n* = 3) Great Britain (*n* = 1)	Healthy adults, adults with health conditions: alcoholism, COPD, chronic stroke, hypertension, cancer, mental-health conditions, psychiatric out- and inpatients, major depression disorder; 1090 (11–92)	IG: forest therapy: walking as a key component in the forest, other therapeutic activities (experiencing the forest through all five senses: seeing, hearing, touching, smelling, tasting), viewing the forest/meditation, Qi-Qong, aromatherapy, herbal tea therapy, handicrafts with natural items CG: normal daily routine; conducted same activities in the room/city area/urban forest/hospital/stay in a hotel; regular diet and exercise program in the forest; one-day trip for urban walking; Durations: 12 min to 3 days for 1 day to 12 weeks
Oh (2017); Australia [19]	5 DBs, from inception to Dec. 2016; GL: was searched LR: English language only	Effects of forest bathing on health; No	RoB SB: high (with 66%) PB: high DB: high (with 30%) AB: unclear or low RB: low	*N* = 6 (6 RCT); China (*n* = 3) Korea (*n* = 2) Sweden (*n* = 1)	Healthy adults, adults with health conditions: chronic alcoholic, high blood pressure, exhaustion disorders, COPD; 323 (18–99)	IG: forest-healing camp (interaction with nature; mountain climbing und trekking; self-introspection, including mediation and counselling); mountain-forest walks; forest-rehabilitation group (with subsequent cognitive behavioural rehabilitation for all participants) CG: normal daily routine; walking/hiking/interventions in urban area/city; waiting list group with cognitive behavioural rehabilitation; Durations: 1 h to 2× week for 11 weeks (22 visits each with 4 h)
Song (2017); South Korea [34]	6 DBs, from inception to Dec. 2016; GL: n. a. LR: English and Korean language only	Effects of forest therapy programs for elementary-school students; No	RoBANS SB: confounder high (with 50%); comparability—predominantly low; selection of participants—low PB: high DB: high AB: predominantly low RB: predominantly low	*N* = 17 (two of them without forest intervention; 10 NRCT, 7 NCT); South Korea	Healthy children; 1491 (16–308)	IG: walks; forest athletic meetings; getting along with neighbours; forest-ecology exploration; making something with natural materials; psychotherapy; climate therapy; exercise therapy; diet therapy; water therapy CG: normal daily routine; traditional learning methods, reduced forest program at school; Durations: 5 h to 8 months
Chae (2018); South Korea [35]	5 DBs, from inception to March 2018; GL: n. a. LR: English and Korean language only	Effects of forest healing therapy; No	n. a.	*N* = 25 (5 of them without forest intervention; 13 NRCT, 12 NCT; Korea	Nurses, healthcare worker, healthy adults, adults with health conditions: alcoholism, Hwa-Byung, depression, mild cognitive impairment, cancer; 1141 (10–221)	IG: nature-experiencing physical activities; forest (healing) program; meditation; hiking CG: interventions conducted indoors; other settings (ambulatory treatment); comparator duration of intervention; no intervention; Durations: 1 day to 12 weeks
Putra (2018); Indonesia[33]	2 DBs, from 2007 to July 2017; GL: n. a. LR: English language only	Effects of phytoncides when forest bathing; No	n. a.	*N* = 10 (n. a.); Japan	Healthy adults; 126 (12–17)	IG: walking, sitting and watching in the forest; physical activity; stay in the hotel for 3 days and 3 nights and giving aromatic volatile substances (phytoncides produced by vaporising *Chamaecyparis obtusa* stem oil) with a humidifier in the hotel room for 3 nights CG: city trips: walking, sitting and watching; normal physical activity; Durations: 4.5 h to 2–3 days
Antonelli, (2019); Italy[27]	6 DBs, from inception to Feb. 2019;GL: was searched LR: English, French, Spanish, and Italian only	Effects of forest bathing on levels of salivary or serum cortisol as stress biomarkers; Yes	NIH; RoB SB: low or unclear PB: high DB: low AB: low or unclear RB: low	*N* = 22 (3 RCT, 8 RCT cross-over, 5 NRCT, 3 NRCT cross-over,3 NCT);Japan (*n* = 12) South Korea (*n* = 4)China (*n* = 2) Germany (*n* = 1) Iceland (*n* = 1), Finland (*n* = 1) Spain (*n* = 1)	Healthy children and adults, adults with health conditions: COPD, high risk of stress/burnout, major depressive disorder, hypertension, post-menopausal women; 2165 (9–348)	IG: forest bathing: spending time in a forest, walking, resting, watching, and deep breathing in forest; psychological program; cognitive behaviour therapy CG: walking and/or watching an urban area/-park; spending time on beach; no intervention; indoor; psychological program; comparator age; Durations: 15 min to half a day
Farrow (2019); USA [30]	2 DBs, from 2008 to 2018; GL: n. a. LR: n. a.	Effects of forest bathing on reducing anxiety and heart rate variability (activation of parasympathetic nervous system); No	n. a.	*N* = 10 (2 RCT, 3 RCT cross-over, 1 NRCT cross-over, 1 NRCT, 3 NCT); Japan (*n* = 8) Taiwan (*n* = 1) Finland (*n* = 1)	Healthy adults, hypertensive adults; 1667 (9–625)	IG: walking, sitting in forest environment, viewing a forest landscape CG: walking, sitting in urban environment, viewing an urban landscape; Durations: 15 mint half a day (4–4.5h); 15 min on 2 days in a row
Wen (2019); China [36]	3 DBs from 2015 to April 2019; GL: was searched LR: English language only	Effects of forest environment exposure on human health; No	Downs and Black Checklist 16 studies high- and 12 studies low-quality; RoB relatively high overall	*N* = 28 (7 RCT, 10 RCT cross-over, 1 NRCT cross-over,3 NRCT, 7 NCT); Japan (*n* = 13) China (*n* = 6) South Korea (*n* = 5) Taiwan (*n* = 3) Poland (*n* = 1)	Healthy children, adults, adults with health conditions: high blood pressure, COPD, chronic stroke, chronic heart failure; 924 (6–128)	IG: exposed to forest (urban forest park), walking, meditation, “five sense experience”, activities and rest, watching the scenery—forest environment, handicrafts, sitting quietly in a dense/sparse forest environment; taking a tree-measuring course; enjoying private time CG: exposed to urban environment/walking and meditating; watching the scenery—urban environment; sitting quietly in a dense/sparse forest environment; indoor classes; Durations: 15 min to 5 days
Kotera (2020); United Kingdom [29]	4 DBs from inception to Oct. 2019; GL: was searched LR: English language only	Effects of Shinrin-Yoku (forest bathing) and nature therapy on mental health; Yes	1. NOS 2. Quality Assessment Table of Randomised Controlled Trials RCTs high to medium; RoB in 6 studies low and in 2 studies high	*N* = 20 (11 RCT, 2 NRCT, 7 NCT); Japan (*n* = 10) Korea (*n* = 4) Taiwan (*n* = 2) Poland (*n* = 2) China (*n* = 1) Serbia (*n* = 1)	Healthy adults, adults with health conditons: metabolic syndrome, chronic stroke, psychiatric disorders (depression), chronic diseases, chronic pain, alcoholism; 2257 (12–585)	IG: Walk in forest and meditation CG: Crossover (forest vs. city); groups with different forest types (birch, maple, and oak); Durations: 15 min to 9 days

AB: attrition bias; CG: control group; COPD: chronic obstructive pulmonary disease; DB: detection bias; DBs: databases; GL: grey literature; IG: intervention group; LR: language restriction; MA: meta-analysis; n. a.: not applicable or not specified; NCT: uncontrolled trial; NIH: National Institutes of Health; NOS: Newcastle–Ottawa Scale; NRCT: non-randomised controlled trial; PB: performance bias; POMS: Profile of Mood States; RB: reporting bias; RCT: randomised controlled trial; RoB: Cochrane Risk-of-Bias tool for randomised controlled trials; RoBANS: Cochrane Risk of Bias Assessment tool for Non-randomised Studies; SB: selection bias; SIGN: The Scottish Intercollegiate Guideline Network measurement tool; SR: systematic review.

**Table 2:** Summary of included reviews and meta-analyses—study populations, indications, and results of the included primary studies, arranged in descending order by methodological quality.

Author	AMSTAR-2 Rating	Study Population	Indications	Outcomes	Results
Ideno, Y. et al. (2017) [28]	moderate	Healthy adults, adults with health conditions: hypertension	Cardiovascular System	Blood pressure: systolic (SBP)/diastolic (DBP)	SBP (20 studies): ↓↓ in forest group DBP (17 studies): ↓↓ in forest group
		Healthy adults, adults with health conditions: hypertension	Cardiovascular system	Heart Rate and Pulse Rate	HR (5 studies)/PR (8 studies): ↓↓ in forest group
Kotera, Y. et al. (2020) [29]	moderate	Healthy adults, adults with health conditions: metabolic syndrome, chronic stroke, psychiatric disorders (depressions), chronic diseases, chronic pains, alcoholism	Mental health	Depression: POMS; BDI; HDR; DASS; EQVAS; MMS Anxiety: POMS; STAI; AAQ; DASS; EQVAS	Depression (20 studies): ↓↓ forest vs. city Anxiety: (5 studies) ↓ after forest intervention, (16 studies) ↓↓ after forest intervention
		Healthy adults, adults with health conditions: metabolic syndrome, depressive tendencies, chronic diseases	Mental health	Anger: POMS	Anger (14 studies): ↓↓ after forest intervention
Lee, I. et al. (2016) [31]	low	Children/adolescents with atopic dermatitis	Atopic dermatitis	Atopic Dermatitis: SCORAD Index (subjective/objective) Child Anxiety-Test Immunology blood test: TARC, MDC,Serum immune cytokine levels	Atopic Dermatitis SCORAD Index (subjective/objective) (all studies): all ↓↓ Child-Anxiety-Test (1 study): ↓ Immunology blood test (1 study): WBC + CD8+ T cells ↑↑; Haemoglobin+ N cells ↓↓, all other ↔; TARC (1 study): ↓; MDC (1 study): ↓↓; serum immune cytokine levels (1 study): IL-5 ↓↓; IL-2, IL-4, IFN-y ↔
Lee, I. et al. (2017) [32]	low	Healthy adults, adults with health conditions: alcoholism, COPD, chronic stroke, hypertension, cancer, mental disorders, psychiatric inpatients or outpatients, severe depression	Mental health	Physical activity: HPLP II Health promotion behaviour: HPLP II Quality of life: EQ-VAS; GHQ/QL-12, SF-36, WHOQOL-BREF, POMS	Physical activity (1 study): ## Health promotion behaviour (1 study): ## Quality of life (5 studies): ## POMS (15 studies): 14 studies ##, 1 study #
		Healthy adults, adults with health conditions: alcoholism, COPD, chronic stroke, hypertension, cancer, mental disorders, psychiatric inpatients or outpatients, severe depression	Mental health	Depression: BDI; MADRS; HDR-D17; Zung Self-Rating Depression Scale Anxiety: STAI	Depression (15 studies): 14 ##, 1 study # Anxiety (6 studies): 5 studies ##, 1 study #
		Healthy adults, adults with health conditions: alcoholism, COPD, chronic stroke, hypertension, cancer, mental disorders, psychiatric inpatients or outpatients, severe depression	Mental health	Stress: Stress response, inventory measuring self-reported stress and arousal Hwa-Byung-Syndrome: The Instrument of Oriental Medical Evaluation for Hwa-Byung	Stress:(2 studies): ## Hwa-Byung-Syndrome (1 study): ##
		Healthy adults, adults with health conditions: alcoholism, COPD, chronic stroke, hypertension, cancer, mental disorders, psychiatric inpatients or outpatients, severe depression	Mental health	Anger: STAXI Emotion: SD -Method	Anger (2 studies): ↔ Emotion (4 studies): 3 studies ##, 1 study #
		Healthy adults, adults with health conditions: alcoholism, COPD, chronic stroke, hypertension, cancer, mental disorders, psychiatric inpatients or outpatients, severe depression	Mental health	Self-esteem: Self-esteem, The Rosenberg Self-Esteem Scale Spirituality: SHI, The Spiritual Assessment Scale Resilience: Self-regulation Resilience Pos./Neg. Effects: PANAS, ROS	Self-esteem (3 studies): ## Spirituality (1 study): ## Resilience (1 study): ## Pos./Neg. Effects (1 study): ##
		Healthy adults, adults with health conditions: alcoholism, COPD, chronic stroke, hypertension, cancer, mental disorders, psychiatric inpatients or outpatients, severe depression	Mental health	NK cells Blood pressure Salivary amylase (Saliva/Serum) Cortisol Pulse rate or Heart rate (PR/HR) HRV/(In LF/HF) Cytokine: IL-6 Enzyme-bonded Immunoassay	NK cells (1 study): ## Blood pressure (6 studies): 3 studies ##, 1 study #, 1 study n.a. Salivary amylase (2 studies): 1 study ##, 1 study # (Saliva/Serum) Cortisol (4 studies): ## PR Or HR (7 studies): 5 studies ##, 1 study #, 1 study n.a. HRV (In LF/HF) (8 studies): 5 studies ##, 2 studies #, 1 study n.a. Cytokine (1 study) IL-6: ## Enzyme-bonded Immunoassay (1 study): ##
Oh, B. et al. (2017) [19]	low	Adults with health conditions: hypertension	Cardiovascular system	Blood pressure Heart function: Cardio Ankle Vascular Index (CAVI)	Blood pressure (2 studies): ↓↓ in forest group Heart function (1 study): ↑↑ in forest group
		Healthy adults, adults with health conditions: COPD	Immune and/or inflammatory indices	Immune function: Expression von Perforin (NK cells, NK-similar cells, CD8 + T cells) and granzyme B Inflammation: Proinflammatory cytokines (IFN-y, IL-6, IL-8, IL-1ß); C-reactive protein (CRP) Inflammation/Tumor: Tumor necrosis factor α	Immune function (1 study): ↓↓ Perforin and granzyme B in forest groupInflammation: Proinflammatory cytokines (3 studies): ↓↓ in forest group; CRP (1 study): ↓ in forest group Inflammation/Tumor (2 studies): 1 study ↓↓ in forest group, 1 study ↔
		Healthy adults	Immune and/or inflammatory indices	Antioxidants: Biomarkers for malondialdehyde (MDA) and superoxide dismutase (SOD)	Antioxidants (1 study): ↓↓ MDA in forest group, SOD ↔
		Older women	Lung function	Lung function: FEV1 (in 1 s) and FEV6 (in 6 s)	Lung function: FEV (in 1s) and FEV (in 6s) (1 study): ↑↑ in forest group
		Adults with chronic health conditions: alcoholism, fatigue syndrome	Mental health	Anxiety and Depression: BDI HADS	Anxiety and Depression: BDI (1 study): ↓↓ forest group HADS (1 study): both groups ↓↓, ↔ between groups
		Adults with health conditions: fatigue syndrome, COPD	Mental health	Stress: PSQserum cortisol	Stress: PSQ (1 study): ↓ in forest group cortisol (1 study): ↓↓ in forest group
		Healthy adults, adults with health conditions: hypertension, COPD	Mental health	Mood: POMS	Mood (3 studies): all ↑↑ in forest group
Song, M. K. and Bang, K. -S. (2017) [34]	low	Healthy children	Mental health	Depression: CDI; Scale for Mental Health Measurement Anxiety: STAI-C; Scale for Mental Health measurement	Depression (4 studies): 2 studies ##, 2 studies # Anxiety (3 studies): 2 studies ##, 1 studies #
		Healthy children	Mental health	Stress: Stress Recognition Inventory for School-aged Children; saliva cortisol level; AMHI	Stress (3 studies): 1 study stress recognition ##; 1 study salivary cortisol ##; 1 study AMHI stress level #
		Healthy children	Mental health	Self-esteem: Self-esteem scale/self-esteem	Self-esteem (3 studies): ##
		Healthy children	Mental health	Negative Emotions: Draw a Story (DAS); scale for emotional and social development test Anger: Novaco Anger Scale Impulsiveness: Scale for mental-health measurement Aggression: Scale for aggression; scale for mental health measurement Attack: Scale for mental health measurement	Negative Emotions (2 studies): ## Anger (1 study): ## Impulsiveness (2 studies): 1 study ##; 1 study # Aggression (2 studies): ## Attack (1 study): #
		Healthy children	Mental health	Mental health: AMHI; scale for mental-health measurement; scale for psychological, social, moral, and physical level	Mental health (4 studies): 2 studies #; 1 study mental health quotient ##; 1 study psychological level ##
		Healthy children	Mental health	Subject Well-being: Subject Well-being Scale	Subject Well-being (1 study): #
		Healthy children	Mental health	Psychological skills: Emotional Intelligence Test; AMHI; Ergo-Resilience Scale	Psychological skills (3 studies): 1 study Ergo-Resilience Scale: interpersonal relationship and curiosity ##.; 1 study emotional intelligence ##; 1 study possibility of problem behaviour (chronic fatigue) and psychological resources ##
		Healthy children	Mental health	Physical skills: Scale for psychological, social, moral, and physical level	Physical skills (1 study): ##
		Healthy children	Sociality	Sociality: Korean Personality Inventory: sociality Social competence: Social Competence Scale; scale for emotional and social development test; scale for psychological, social, moral, and physical level	Sociality (2 studies): ## Social competence (3 studies): ##
		Healthy children	Sociality	School violence attitude: Scale for school violence attitude School adjustment: Scale for school adjustment; multiple life satisfaction scaleLife respect: Scale for life respect	School violence attitude (1 study): ## School adjustment: (2 studies): both in parts ## Life respect (1 study): ##
Antonelli, M. et al. (2019) [27]	low	Healthy children, adults with health conditions: COPD, high risk for stress/burnout, severe depression, hypertension, postmenopausal women	Mental health	Stress: salivary/serum cortisol	Stress (22 studies): 20 studies ↓↓ forest vs. non-forest within group: 13 studies ↓↓, 1 study ↑↑, 5 n.a. between groups: 13 studies ↓↓ forest vs. non-forest, 1 ↑↑ forest vs. non-forest, 4 ↔, 3 n. a.
Wen, Y. et al. (2019) [36]	low	Healthy children/adults, adults with health conditions: hypertension, COPD, chronic stroke, chronic heart failure	Cardiovascular system	Blood pressure: systolic/diastolic Heart rate Pulse rate HRV	Blood pressure (8 studies): ↓↓ in 5 studies in forest group, 1 study ↓↓ between groups, 1 study ↑↑ between groups, 1 study irregular index Heart rate (3 studies): all ↓↓ between groups Pulse rate (5 studies): ↓↓ in 4 studies, 1 study ↓; HRV (6 studies): lnHF ↑↑ in 4 studies, 1 study ↓; lnLF/lnHF ↓↓ in 2 studies, 1 study ↓, 2 studies ↑
		Healthy adults, adults with health conditions: hypertension	Metabolic parameters	Triglyceride Adiponectin	Triglyceride (2 studies): ↓↓ Adiponectin (1 study): ↑↑
		Healthy adults, adults with health conditions: hypertension, COPD, chronic heart failure	Immune and/or inflammatory indices	NK cells NKT cells Interleukin (IL-6, IL-8) Tumor necrosis factor CRP	NK cells (2 studies): ↓↓ NKT cells (1 study): ↓↓; Interleukin (4 studies): in 3 studies ↓↓ Tumor necrosis factor (3 studies): 2 studies ↓↓ CRP (3 studies): 1 study ↓↓
		Adults with health conditions: chronic heart failure	Immune and/or inflammatory indices	Antioxidants: Glutathione peroxidase biological antioxidant potential; peroxides (8-hydroxy-2’-deoxyguanosine and hydrogen); MDAHyperventilation	Antioxidants: Glutathione peroxidase (1 study): ↑↑, biological antioxidant potential (1 study): ↑↑, Peroxides (1 study): ↓↓, MDA (2 studies): ↓↓ Hyperventilation (2 studies): ↓↓
		Healthy adults, adults with health conditions: hypertension, COPD	Mental health	Stress: Stress management; stress reaction; cortisol; adrenaline; norepinephrine; dopamine	Stress (6 studies): management 1 study ↑↑ between groups; reaction 1 study ↓↓ between groups; hormones (5 studies): 2 studies ↓↓ within group, 3 studies ↓↓ between groups
		Healthy adults	Mental health	Electrophysiological indices:EEG, high alpha brain waves and high beta brain waves	Electrophysiological indices (1 study): ↑↑
		Healthy adults, adults with health conditions: hypertension, COPD, chronic heart failure	Mental health	POMS	POMS (14 studies): in 11 studies ↓↓ negative emotions, 10 studies ↑↑ tension, 2 studies ↑↑ positive emotions
		Healthy adults, adults with health conditions: hypertension	Mental health	Attitudes and feelings about things: SD method	Attitudes and feelings about things (6 studies): in 5 studies ↑↑; 1 study n.a.
		Healthy children/adults, adults with health conditions: chronic stroke	Mental health	Anxiety and Depression: BDI, HDR, CDI, STAI	Anxiety (6 studies): 4 studies ↓↓ within group, 3 studies ↓↓ between groups Depression (3 studies): 2 studies ↓↓ within group, 2 studies ↓↓ between groups
		Healthy adults	Mental health	Degree of phys./psych. regeneration: Somatic Quality of Life: EQ-5D-3L; somatic symptoms; psychological regeneration; mental health	Degree of phys./psych. regeneration: Somatic Quality of life (1 study): ↑↑ within group somatic symptoms (2 studies): ↓↓ psychological regeneration, mental health (2 studies): ↑↑
		Healthy children/adults	Mental health	Adaptive Behaviour:Self-esteem; health-promoting behaviour	Adaptive Behaviour: self-esteem (1 study): ↑↑ health-promoting behaviour (1 study): ↑↑
Chae, Y. R. et al. (2018) [35]	critically low	Healthy adults	Metabolic parameters	Triglycerid Level	Triglycerid Level (1 study): ↓↓
		Healthy adults	Menopausal symptoms	Menopausal Complaints: Menopausal symptoms	Menopausal Complaints: Menopausal symptoms (1 study): ↓
		Healthy adults: adults with health conditions: alcohol dependency	Mental health	Stress: Employment/job stress; psychosocial stress	Stress (8 studies): all ↓
		Healthy adults: adults with health conditions: alcohol dependency; Hwa-Byung; depression; cancer; mild cognitive impairment	Mental health	Depression: BDI; HDR; MADRS Anxiety: n.a.	Depression (9 studies): all ↓ Anxiety (7 studies): 4 studies ↓, 2 studies ↔
		Healthy adults: adults with health conditions: Hwa-Byung; depression; mild cognitive impairment	Quality of Life	Quality of life: SF-36; QoL	Quality of life (4 studies): all ↑
		Healthy adults: adults with health conditions: alcohol dependency	Mental health	Emotion: POMS	Emotion (2 studies): ↑↑
		Healthy adults: adults with health conditions: Hwa-Byung; depression; mild cognitive impairment	Mental health	Heart Rate Variability (HRV)	HRV (2 studies): ↑
Putra, R. et al. (2018) [33]	critically low	Healthy adults	Immune and/or inflammatory indices	Immune function: NK activity; adrenaline concentration in urine; salivary/serum cortisol Blood pressure Pulse rate (PR) HRV WBC POMS	Immune function: NK activity (4 studies): all ↑↑ in forest group, adrenaline (4 studies): 2 studies ↓↓ in forest group, 1 study ↔, 1 study ↑↑; cortisol (5 studies): 3 studies ↓↓, 1 study ↔ in forest group, 1 study ↓ in both groups Blood pressure (4 studies): 1 study ↔, 2 studies ↓↓ in forest group, 1 study n.a. Pulse rate (5 studies): 3 studies ↓↓, 1 study unchanged, 1 study ↑↑ in forest group HRV (3 studies): 1 study ↑↑, 1 study ↓↓, 1 study ↔ in forest group WBC (4 studies): 2 studies ↔, 2 studies n. a. POMS (4 studies): all ↑↑ in forest group
Farrow, M. R. and Washburn, K. (2019) [30]	critically low	Healthy adults	Mental health	Activity of the parasympathetic nervous system: HRV (InLF/HF, InHF)	Activity of the parasympathetic nervous system: ↑↑ InHF, ↓↓ lnLF/HF
		Healthy adults, adults with health conditions: hypertension	Mental health	Blood pressure Pulse rate Salivary cortisol level Dopamine/Adrenaline/Noradrenalin in Urine Anxiety (POMS subscale values, SD Method)	Blood pressure (2 studies): ↓ Pulse rate (6 studies): ↑ Salivary cortisol (3 studies): ↓ Adrenaline in urine (1 study): ↓ Anxiety (8 studies): ↓

↓↓ significantly reduced; ↓ reduced, not significant; ↑↑ significant improvement; ↑ improvement, not significant; ↔ unchanged; ## significant change—not specified by authors; # change, not significant—not specified by authors; AAQ: Anti-Anxiety Questionnaire; AMHI: Adolescent Mental Health Inventory; BDI: Beck Depression Inventory; CDI: Children’s Depression Inventory; COPD: chronic obstructive pulmonary disease; CRP: C-reactive protein; DAS: Draw a Story; DASS: Depression Anxiety and Stress Scale; DBP: diastolic blood pressure; EEG: electroencephalogram; EQVAS: EuroQol Visual Analog Scale; EQ-5D-3L: European Quality of Life 5 Dimensions 3 Level Version; GHQ/QL-12: General Health Questionnaire/Quality of Life; HADS: Hospital Anxiety and Depression Scale; HDR: Hamilton Depression Rating Scale; HPLP II: Health-Promoting Lifestyle Profile II; HR: heart rate; HRV: heart rate variability; Hwa-Byung: Korean culture-induced anger syndrome with symptoms of insomnia, depression, and somatisation in the lower abdomen; MADRS: Montgomery–Åsberg Depression Rating Scale; MDA: malondialdehyde biomarkers; MDC: macrophage-derived chemokine; MMS: Multiple Mood Scale; NK cells: natural killer cells; PANAS: Positive and Negative Affect Schedule; POMS: Profile of Mood States; PR: pulse rate; ROS: The Restorative Outcome Scale; SBP: systolic blood pressure; SCORAD: Scoring Atopic Dermatitis; SD Method: Semantic Differential Method; SF-36: short form health 36; SHI: Spiritual Health Inventory; SMHM: Scale for Mental Health Measurement; SOD: superoxide dismutase; STAI: The Spielberger State–Trait Anxiety Inventory; STAXI (-C): State–Trait Anger Expression Inventory (for children); TARC: thymus and activation-regulated chemokine; WBC: white blood cell count; WHOQOL-BREF: WHO Quality of Life–BREF.

**Table 3:** Summary of associations between forest-based interventions and health effects/outcomes on the cardiovascular system.

	Health Effects/Outcomes	Forest-Based Interventions	Reference of Association (+)	Reference of Association (+/−)
Cardiovascular system	Blood pressure	Walking/sitting in and viewing forest area	Ideno, Y. et al. (2017) [28]	
		Walking in forest area	Oh, B. et al. (2017) [19]	
		Walking/activity/rest in forest area	Wen, Y. et al. (2019) [36]	
	Heart and pulse rate	Walking/activity/rest in forest area	Wen, Y. et al. (2019) [36]	
		Sitting in and viewing forest area/walking and activities in the forest	Ideno, Y. et al. (2017) [28]	
	Cardio Ankle Vascular Index (CAVI)	Walking in forest area	Oh, B. et al. (2017) [19]	
	HRV	Walking/activity/rest in forest area	Wen, Y. et al. (2019) [36]	

HRV: heart rate variability; +: mainly an improvement; +/−: mixed results.

**Table 4:** Summary of associations between forest-based interventions and health effects/outcomes in immune and inflammatory parameters.

	Health Effects/ Outcomes	Forest-Based Interventions	Reference of Association (+)	Reference of Association (+/−)
Immune and/orinflammatory parameters	NK/NKT cells	Walking in forest area	Oh, B. et al. (2017) [19]	
		Walking in forest area	Wen, Y. et al. (2019) [36]	
	Cytokines	Walking in forest area	Oh, B. et al. (2017) [19]	
		Walking in forest area; exposed to forest	Wen, Y. et al. (2019) [36]	
	CRP	Walking in forest area	Oh, B. et al. (2017) [19]	
		Walking in forest area	Wen, Y. et al. (2019) [36]	
	Tumour necrosis factor	Walking in forest area		Oh, B. et al. (2017) [19]
		Walking in forest area; exposed to forest	Wen, Y. et al. (2019) [36]	
	Antioxidants	Walking in forest area		Oh, B. et al. (2017) [19]
		Walking and meditation in forest area; exposed to forest	Wen, Y. et al. (2019) [36]	

CRP: C-reactive protein; NK cells: natural killer cells/NKT cells: natural killer T-cells; +: mainly improvement; +/−: mixed results.

**Table 5:** Summary of associations between forest-based interventions and health effects/outcomes in metabolic parameters.

	Health Effects/ Outcomes	Forest-Based Interventions	Reference of Association (+)	Reference of Association (+/–)
Metabolic Parameters	Triglyceride level	Walking in forest area	Wen, Y. et al. (2019) [36]	
	Adiponectin level	Walking in forest area	Wen, Y. et al. (2019) [36]	

+: mainly improvement; +/–: mixed results.

**Table 6:** Summary of associations between forest-based interventions and health effects/outcomes in atopic dermatitis.

	Health Effects/ Outcomes	Forest-Based Interventions	Reference of Association (+)	Reference of Association (+/–)
Skin Diseases (Atopic Dermatitis)	SCORAD Index	Forest experience, forest trip; forest activity; forest camp; forest camp swimming	Lee, I. et al. (2016) [31]	
		Forest trip; forest activity; forest camp		Lee, I et al. (2016) [31]

SCORAD Index: Scoring Atopic Dermatitis Index; +: mainly improvement; +/–: mixed results.

**Table 7:** Summary of associations between forest-based interventions and health effects/outcomes in mental health.

	Health Effects/ Outcomes	Forest-Based Interventions	Reference of Association (+)	Reference of Association (+/−)
Mental Health	Stress(cortisol level/PROs)	Walking in forest area; forest healing program	Lee, I. et al. (2017) [32]	
		Forest healing program (with cognitive behavioural rehabilitation)	Oh, B. et al. (2017) [19]	
		Forest activity; forest healing program	Song, M. K. and Bang, K. -S. (2017) [34]	
		Walking/activity/rest in forest area	Wen, Y et al. (2019) [36]	
		Forest bathing: spending time in a forest, walking, resting, watching, and deep breathing for a limited time in forest	Antonelli, M. et al. (2019) [27]	
	Anxiety and depression (PROs)	Walking in forest area, resting in the forest, forest healing program, forest therapy	Lee, I. et al. (2017) [32]	
		Forest healing program (with cognitive behavioural rehabilitation)	Oh, B. et al. (2017) [19]	
		Activity in forest area, forest experience program		Song, M. K. and Bang, K. -S. (2017) [34]
		Walking/activity/mediation/rest in forest area	Wen, Y. et al. (2019) [36]	
		Waking, mediation in forest area, forest therapy program	Kotera, Y. et al. (2020) [29]	
		Walking, activity, resting in forest area, forest experience program	Lee, I. et al. (2017) [32]	
	Negative emotions(PROs)	Walking in forest area	Oh, B. et al. (2017) [19]	
		Activity in forest area, forest experience program, forest ecology exploration	Song, M. K. and Bang, K. -S. (2017) [34]	
		Walking/activity/rest in forest area	Wen, Y. et al. (2019) [36]	
		Walking, resting/breathing in forest area, recreation program, forest therapy program, forest bathing program	Kotera, Y. et al. (2020) [29]	
	Quality of life/well-being(PROs)	Walking in the forest; forest-healing program, forest therapy	Lee, I. et al. (2017) [32]	
		Activities in the forest		Song, M. K. and Bang, K. -S. (2017) [34]
		Walking/activities in the forest	Wen, Y. et al. (2019) [36]	

PRO: patient reported outcome; +: mainly improvement; +/−: mixed results.

**Table 8:** Summary of associations between forest-based interventions and health effects / outcomes in sociality.

	Health Effects / Outcomes	Forest-based Interventions	Reference of Association (+)	Reference of Association (+/–)
Sociality	Sociality(PRO)	Forest-experience program, forest-therapy program	Song, M. K. & Bang, K.-S. (2017) [32]	
	Social Competence (PROs)	Forest-healing program, ecology exploration	Song, M. K. & Bang, K.-S. (2017) [32]	
	School Violence Attitude (PRO)	Forest-ecology exploration	Song, M. K. & Bang, K.-S. (2017) [32]	
	School Adjustment (PROs)	Activities in the forest; forest-experience program		Song, M. K. & Bang, K.-S. (2017) [32]
	Life Respect (PRO)	Forest-therapy program	Song, M. K. & Bang, K.-S. (2017) [32]	

PRO: Patient Reported Outcome; +: mainly improvement; +/–: mixed results.

## Appendix A. Search Strings

**Table A1:** Medline.

No.	Search Strings	Hits
1	“forest therap*”.ab,ti.	30
2	“forest bath*”.ab,ti.	27
3	forest medicine.ab,ti.	4
4	Shinrin-yoku.ab,ti.	18
5	forest healing.ab,ti.	1
6	“review*”.ab,ti.	1,732,382
7	“systematic*”.ab,ti.	360,076
8	“overview*”.ab,ti.	133,772
9	“Meta-Analys*”.ab,ti.	133,384
10	1 or 2 or 3 or 4 or 5	63
11	6 or 7 or 8 or 9	2,052,748
12	10 and 11	12

**Table A2:** Embase.

No.	Search Strings	Hits
1	“forest therap*”.ab,ti.	39
2	“forest bath*”.ab,ti.	45
3	forest medicine.ab,ti.	4
4	Shinrin-yoku.ab,ti.	21
5	forest healing.ab,ti.	4
6	“review*”.ab,ti.	2,679,607
7	“systematic*”.ab,ti.	565,832
8	“overview*”.ab,ti.	198,810
9	“Meta-Analys*”.ab,ti.	217,515
10	1 or 2 or 3 or 4 or 5	90
11	6 or 7 or 8 or 9	3,173,197
12	10 and 11	22

**Table A3:** Web of Science.

No.	Search Strings	Hits
# 12	#11AND#10	64
# 11	#9OR#8OR#7OR#6	3,453,809
# 10	#5OR#4OR#3OR#2OR#1	470
# 9	TOPIC: (Meta-Analys*)	196,262
# 8	TOPIC: (overview*)	331,638
# 7	TOPIC: (systematic*)	889,313
# 6	TOPIC: (review*)	2,509,712
# 5	TOPIC: (forest healing)	287
# 4	TOPIC: (“Shinrin-yoku”)	112
# 3	TOPIC: (“forest medicine”)	3
# 2	TOPIC: (“forest bath*”)	70
# 1	TOPIC: (“forest therap*”)	68

**Table A4:** Cochrane Library.

No.	Search Strings	Hits
#1	(review*):ti,ab,kw OR (systematic*):ti,ab,kw OR (overview*):ti,ab,kw OR (Meta-Analys*):ti,ab,kw (Word variations have been searched)	91,743
#2	(forest NEXT therap*):ti,ab,kw OR (forest NEXT bath*):ti,ab,kw OR (Forest NEXT medicine):ti,ab,kw OR (Shinrin-Yoku):ti,ab,kw OR (forest NEXT healing):ti,ab,kw (Word variations have been searched)	27
#3	#1 AND #2	0

**Table A5:** Psycinfo.

No.	Search Strings	Hits
S3	(S1 AND S2)	2
S2	review* OR systematic* OR overview* OR Meta-Analys*	776,703
S1	“forest therap*” OR “forest bath*” OR “forest medicine” OR “shinrin-yoku” OR “forest healing”	12

**Table A6:** EBSCO.

No.	Search Strings	Hits
S3	(S1 AND S2)	2
S2	review* OR systematic* OR overview* OR Meta-Analys*	776,703
S1	“forest therap*” OR “forest bath*” OR “forest medicine” OR “shinrin-yoku” OR “forest healing”	12

**Table A7:** CiNii.

No.	Search Strings	Hits
1	(forest therap* OR forest bath* OR forest medicine OR Shinrin-Yoku OR forest healing) AND (review* OR systematic* OR overview* OR Meta-Analys*)	21

**Table A8:** Scopus.

No.	Search Strings	Hits
1	(TITLE-ABS-KEY(“forest therap*” OR “forest bath*” OR “forest medicine” OR “shinrin-yoku” OR “forest healing”)AND TITLE-ABS-KEY(review* OR systematic* OR overview* OR meta-analys*))	31

**Table A9:** Google Scholar.

No.	Search Strings	Hits
1	review AND [“forest therapy” OR “forest bathing” OR “forest medicine” OR “shinrin-yoku” OR “forest healing” -doi]	1090

**Table A10:** PROSPERO.

No.	Search Strings	Hits
1	(“forest therap*” or “forest bath*” or “forest medicine” or “shinrin-Yoku” or “forest healing”) AND (review* or systematic* or overview* or “Meta-Analys*”)	8

## Appendix B

**Table A11:** Excluded references after full text analysis.

Reference	Reason for Exclusion
Ambrose-Oji, B. Mindfulness Practice in Woods and Forests: An Evidence Review. Research Report for The Mersey Forest, Forest Research. Alice Holt Lodge Farnham, Surrey; 2013. Available from: https://infta.net/files/references/Mindfulness%20and%20Woods%20Evidence%20Review%20%202013.pdf [Access Date: 6 October 2020].	No systematic review
Cho, K. S.; Lim, Y. R.; Lee, K.; Lee, J.; Lee, J. H.; Lee, I. S. Terpenes from Forests and Human Health. Toxicol. Res. 2017;33(2):97–106.	No systematic review
Cho, Y.-M.; Kim, D.-J.; Lee, K. H.; Lee, H. E.; Lee, Y. J. A Study on Effect of Forest Related Programs based on the Meta-Analysis. Journal of the Korean Institute of Forest Recreation, 2015. 19(1): 1–13.	No systematic review
Karjalainen, E., Sarjala, T. & Raitio, H. Promoting human health through forests: overview and major challenges. Environ Health Prev Med. 2010;15:1–8.	No systematic review
Li, Q. Effect of forest bathing trips on human immune function. Environ Health Prev Med. 2010;15:9–17.	No systematic review
Li, Q. Effect of forest bathing (shinrin-yoku) on human health: A review of the literature. Santé Publique. 2019;31:135–143.	No systematic review
Li, Q.; Kawada, T. Possibility of Clinical Applications of Forest Medicine. Japanese Journal of Hygiene. 2014;69(2):117–121.	No systematic review
Meyer, K.; Burger-Arndt, R. How forests foster human health—Present state of research-based knowledge (in the field of Forests and Human Health). International Forestry Review. 2014;16(4):421–446.	No systematic review
Meyer-Schulz, K.; Burger-Arndt, R. Une revue de la littérature scientifique Reviewing the psychological and physical health effects of forests. Santé Publique. 2019; 31:115–134.	No systematic review
Miyazaki, Y.; Ikei, H.; Song, C. Forest medicine research in Japan. Japanese Journal of Hygiene. 2014; 69(2):122–135.	No systematic review
Payne, M.; Delphinus, E. The most natural of natural therapies: A review of the health benefits derived from Shinrin-Yoku (Forest Bathing). The International Journal of Health, Wellness, and Society. 2019;9:19–30.	No systematic review
Shin, W. S.; Yeoun, P. S.; Yoo, R. W.; Shin, C. S. Forest experience and psychological health benefits: The state of the art and future prospect in Korea. Environ Health Prev Med. 2010; 15:38–47.	No systematic review
Zhang, Z. Y.; Wang, P.; Gao, Y.; Ye, B. Current Development Status of Forest Therapy in China. Healthcare (Basel). 2020;8(1):61.	No systematic review
Corazon, S. S.; Sidenius, U.; Poulsen, D. V.; Gramkow, M. C.; Stigsdotter, U. K. Psycho-Physiological Stress Recovery in Outdoor Nature-Based Interventions: A Systematic Review of the Past Eight Years of Research. Int. J. Environ. Res. Public Health. 2019; 16, 1711.	Non-predominant forest (≥80% of the studies)
Haluza, D.; Schonbauer, R.; Cervinka, R. Green Perspectives for Public Health: A Narrative Review on the Physiological Effects of Experiencing Outdoor Nature. Int. J. Environ. Res. Public Health. 2014;11,:5445–5461.	Non-predominant forest (≥80% of the studies)
Hansen, M. M.; Jones, R.; Tocchini, K. Shinrin-Yoku (Forest Bathing) and Nature Therapy: A State-of-the-Art Review. Int. J. Environ. Res. Public Health. 2017;14 (8):851.	Non-predominant forest (≥80% of the studies)
Hossain, M., Sultana, A., Ma, P., Fan, Q., Sharma, R., Purohit, N., & Sharmin, D. F. (2020, January 8). Effects of natural environment on mental health: an umbrella review of systematic reviews and meta-analyses. Available from: https://psyarxiv.com/4r3mh/ [Access Date: 6 October 2020].	Non-predominant forest (≥80% of the studies)
Kamioka H, Tsutani K, Mutoh Y, Honda T, Shiozawa N, Okada S, Park SJ, Kitayuguchi J, Kamada M, Okuizumi H, Handa S. A systematic review of randomized controlled trials on curative and health enhancement effects of forest therapy. Psychol Res Behav Manag. 2012;5:85–95.	Non-predominant forest (≥80% of the studies)
Poulsen, D. V. (2015). How war veterans with post-traumatic stress disorder experience nature-based therapy in a forest therapy garden. Københavns Universitet: Department of Geosciences and Natural Resource Management, Faculty of Science, University of Copenhagen. Available from: https://ign.ku.dk/phd-forsvar/2015/phd-defence-dorthe-varning-poulsen/ [Access Date: 6 October 2020].	Non-predominant forest (≥80% of the studies)
Kim T, Song B, Cho KS, Lee IS. Therapeutic Potential of Volatile Terpenes and Terpenoids from Forests for Inflammatory Diseases. *Int J Mol Sci*. 2020;21(6):2187.	Non-predominant forest (≥80% of the studies)
Mathias, S., Daigle, P., Dancause, K. N., & Gadais, T. (2019, December 5). Forest bathing: a narrative review of the effects on health for outdoor and environmental education use in Canada. Available from: https://doi.org/10.31236/osf.io/j6mq3 [Access Date: 6 October 2020].	Non-predominant forest (≥80% of the studies)
McMahan, E. A.; Estes, D. The effect of contact with natural environments on positive and negative affect: A meta-analysis. The Journal of Positive Psychology. 2015;10(6): 507–519.	Non-predominant forest (≥80% of the studies)
Meredith Genevive R., Rakow Donald A., Eldermire Erin R. B., Madsen Cecelia G., Shelley Steven P., Sachs Naomi A. Minimum Time Dose in Nature to Positively Impact the Mental Health of College-Aged Students, and How to Measure It: A Scoping Review. 2020;10:2942 Available from: https://doi.org/10.3389/fpsyg.2019.02942 [Access Date: 6 October 2020].	Non-predominant forest (≥80% of the studies)
Muranen, Anni-Tuulia; Alihanka, Karoliina. Green Exercise in Supportin the Mental Health of Cardiac patients. Thesis. 2019. Available from: https://www.theseus.fi/bitstream/handle/10024/265719/Muranen_Anni-Tuulia%2C%20Alihanka_Karoliina.pdf?sequence=2&isAllowed=y [Access Date: 6 October 2020].	Non-predominant forest (≥80% of the studies)
Mygind L, Kjeldsted E, Hartmeyer R, et al.. Effects of Public Green Space on Acute Psychophysiological Stress Response: A Systematic Review and Meta-Analysis of the Experimental and Quasi-Experimental Evidence. *Environment and Behavior*. 2019.	Non-predominant forest (≥80% of the studies)
Mygind L, Kjeldsted E, Hartmeyer RD, Mygind E, Bølling M, Bentsen P. Immersive Nature-Experiences as Health Promotion Interventions for Healthy, Vulnerable, and Sick Populations? A Systematic Review and Appraisal of Controlled Studies. *Front Psychol*. 2019;10:943.	Non-predominant forest (≥80% of the studies)
Nilsson K, Bentsen P, Grahn P, Mygind L. De quelles preuves scientifiques disposons-nous concernant les effets des forêts et des arbres sur la santé et le bien-être humains ? [What is the scientific evidence with regard to the effects of forests, trees on human health and well-being?]. Sante Publique. 2019;S1(HS):219–240. French.	Non-predominant forest (≥80% of the studies)
Roberts H, van Lissa C, Hagedoorn P, Kellar I, Helbich M. The effect of short-term exposure to the natural environment on depressive mood: A systematic review and meta-analysis. Environ Res. 2019;177:108606.	Non-predominant forest (≥80% of the studies)
Roth, A.-S. (2011). Naturen—En resurs för stressåterhämtning: En systematisk litteraturstudie (Dissertation). Available from: http://urn.kb.se/resolve?urn=urn:nbn:se:his:diva-5342 [Access Date: 6 October 2020].	Non-predominant forest (≥80% of the studies)
Sun, Seung-Ho; Lee, Seon-Goo. Systemic review on forest healing journals. Korean J. Oriental Physiology & Pathology 2010; 24(4): 566–570.	Non-predominant forest (≥80% of the studies)
Twohig-Bennett, Caoimhe; Jones, Andy. The health benefits of the great outdoors: A systematic review and meta-analysis of greenspace exposure and health outcomes. Environmental Research. 2018, 166:628–637.	Non-predominant forest (≥80% of the studies)
Vibholm, A. P.; Christensen, J. R.; Pallesen, H. Nature-based rehabilitation for adults with acquired brain injury: a scoping review. International Journal of Environmental Health Research, 30:6, 661–676.	Non-predominant forest (≥80% of the studies)

## Appendix C. Deviation from the AMSTAR-2 Guidance Document

We have made the following adjustments for the quality assessment of the current SRs and MAs:

1. Many items in the AMSTAR-2 can be answered with “Partial Yes” or “Yes”. Several criteria must usually be met for a “Partial Yes”. The criteria for “Partial Yes” and several additional criteria must be met for a “Yes”. This is usually formulated as “For Yes should also have...” or “For Yes must also have...”. We have interpreted and applied these rules as follows: for a formulation with “should”, at least one of the following criteria had to be met for a “Yes”, and all the criteria listed had to be met for a formulation with “must”.
2. Item 4: “Did the review authors use a comprehensive literature search strategy?” Three criteria must be met for a “Partial Yes” to this question. One of them is “justified publication restrictions (e.g., language)”. Since the restrictions for the literature search are usually described, but only very rarely justified, we did not take this criterion into account in the evaluation.
3. Item 7: “Did the review authors provide a list of excluded studies and justify the exclusions?” For a “Yes” to this question, a list of the excluded studies must be available, and the exclusion of every potentially relevant study from the review must be justified. The evaluation was modified as follows: for a “Yes”, it was sufficient if the number of excluded studies and information about how many studies had been excluded for what reasons in the paper (e.g., in PRISMA Flow Chart) were listed.

## References

[1] United Nations Department of Economic and Social Affairs (UN/DESA): World Urbanization Prospects: The 2018 Revision. https://population.un.org/wup/Publications/Files/WUP2018-KeyFacts.pdf.

[2] Corazon S.S., Sidenius U., Poulsen D.V., Gramkow M.C., Stigsdotter U.K. (2019). Psycho-Physiological Stress Recovery in Outdoor Nature-Based Interventions: A Systematic Review of the Past Eight Years of Research. Int. J. Environ. Res. Public Health.

[3] Hansen M.M., Jones R., Tocchini K. (2017). Shinrin-yoku (forest bathing) and nature therapy: A state of the art review. Int. J. Environ. Res. Public Health.

[4] Twohig-Bennett C., Jones A. (2018). The health benefits of the great outdoors: A systematic review and meta-analysis of greenspace exposure and health outcomes. Environ. Res..

[5] Bowler D.E., Buyung-Ali L.M., Knight T.M., Pullin A.S. (2010). A systematic review of evidence for the added benefits to health of exposure to natural environments. BMC Public Health.

[6] Li Q. (2010). Effect of forest bathing trips on human immune function. Environ. Health Prev. Med..

[7] Markov S., Steckenbauer G.C., Pillmayer M., Herntrei M., Kotte D., Li Q., Shin W.S., Michalsen A. (2019). Austria: The Forest as a Touristic Landscape. International Handbook of Forest Therapy.

[8] Li Q. (2012). Forest Medicine—Public Health in the 21st Century.

[9] Kotte D., Li Q., Shin W.S., Michalsen A. (2019). International Handbook of Forest Therapy.

[10] Li Q. (2013). What is Forest Medicine?. Forest Medicine.

[11] Schuh A., Immich G. (2019). Waldtherapie—Das Potential des Waldes für Ihre Gesundheit.

[12] Kaiserbaeder Usedom (2002). Cure and Healing Forests. https://www.kur-und-heilwald.de/?lang=en.

[13] Karim A., Khalil R., Schmitt M. (2020). Wald reloaded—Die Neuentdeckung des Waldes aus gesundheitspsychologischer Sicht. Z. Komplementärmedizin.

[14] Vukin M., Isailović G. A Cure and Healing Foest of Goč Mountain—A New Approach to Health Tourism in Serbia. https://de.calameo.com/read/00586528821d0b12eaa9b.

[15] Berman M.G., Jonides J., Kaplan S. (2008). The cognitive benefits of interacting with nature. Psychol. Sci..

[16] Peen J., Schoevers R.A., Beekman A.T., Dekker J. (2010). The current status of urban-rural differences in psychiatric disorders. Acta Psychiatr. Scand..

[17] Van Den Berg A.E., Maas J., Verheij R.A., Groenewegen P.P. (2010). Green space as a buffer between stressful life events and health. Soc. Sci. Med..

[18] Müllner M., Zwick H. (2007). Epidemiologie der Zivilisationskrankheiten. Bewegung als Therapie: Gezielte Schritte zum Wohlbefinden.

[19] Oh B., Lee K.J., Zaslawski C., Yeung A., Rosenthal D., Larkey L., Back M. (2017). Health and well-being benefits of spending time in forests: Systematic review. Environ. Health Prev. Med..

[20] Vibholm A.P., Christensen J.R., Pallesen H. (2020). Nature-based rehabilitation for adults with acquired brain injury: A scoping review. Int. J. Environ. Health Res..

[21] Meyer K., Burger-Arndt R. (2014). How forests foster human health—Present state of research-based knowledge (in the field of Forests and Human Health). Int. For. Rev..

[22] Meyer-Schulz K., Burger-Arndt R. (2019). Reviewing the Psychological and Physical Health Effects of Forests. Santé Publique.

[23] Stier-Jarmer M., Throner V., Kirschneck M., Frisch D., Schuh A. (2020). Psychological and Physical Effects of Forests on Human Health—A Systematic Review of Systematic Reviews and Meta-Analyses.

[24] Stern C., Jordan Z., McArthur A. (2014). Developing the Review Question and Inclusion Criteria. Am. J. Nurs..

[25] Shea B.J., Reeves B.C., Wells G., Thuku M., Hamel C., Moran J., Moher D., Tugwell P., Welch V., Kristjansson E. (2017). AMSTAR 2: A critical appraisal tool for systematic reviews that include randomised or non-randomised studies of healthcare interventions, or both. BMJ.

[26] Cochrane Deutschland Stiftung, Institut für Evidenz in der Medizin, Institut für Medizinische Biometrie und Statistik, Freiburg, Arbeitsgemeinschaft der Wissenschaftlichen Medizinischen Fachgesellschaften, Institut für Medizinisches Wissensmanagement, Ärztliches Zentrum für Qualität in der Medizin (2019). Manual Systematische Recherche für Evidenzsynthesen und Leitlinien. https://freidok.uni-freiburg.de/data/149324.

[27] Antonelli M., Barbieri G., Donelli D. (2019). Effects of forest bathing (shinrin-yoku) on levels of cortisol as a stress biomarker: A systematic review and meta-analysis. Int. J. Biometeorol..

[28] Ideno Y., Hayashi K., Abe Y., Ueda K., Iso H., Noda M., Lee J.S., Suzuki S. (2017). Blood pressure-lowering effect of Shinrin-yoku (Forest bathing): A systematic review and meta-analysis. BMC Complement. Altern. Med..

[29] Kotera Y., Richardson M., Sheffield D. (2020). Effects of Shinrin-Yoku (Forest Bathing) and Nature Therapy on Mental Health: A Systematic Review and Meta-Analysis. Int. J. Ment. Health Addict..

[30] Farrow M.R., Washburn K.A. (2019). Review of Field Experiments on the Effect of Forest Bathing on Anxiety and Heart Rate Variability. Glob. Adv. Health Med..

[31] Lee I., Bang K.S., Kim S., Choi H., Lee B., Song M. (2016). Effect of Forest Program on Atopic Dermatitis in Children—A Systematic Review. J. Korean Inst. For. Recreat..

[32] Lee I., Choi H., Bang K.S., Kim S., Song M., Lee B. (2017). Effects of forest therapy on depressive symptoms among adults: A systematic review. Int. J. Environ. Res. Public Health.

[33] Putra R.R.F.A., Veridianti D.D., Nathalia E., Brilliant D., Rosellinny G., Suarez C.G., Sumarpo A. (2018). Immunostimulant Effect from Phytoncide of Forest Bathing to Prevent the Development of Cancer. Adv. Sci. Lett..

[34] Song M.K., Bang K.-S. (2017). A systematic review of forest therapy programs for elementary school students. Child Health Nurs. Res..

[35] Chae Y.R., Kim J.H., Kang H. (2018). Literature Review of Forest Healing Therapy on Korean Adults. J. Korean Biol. Nurs. Sci..

[36] Wen Y., Yan Q., Pan Y., Gu X., Liu Y. (2019). Medical empirical research on forest bathing (Shinrin-yoku): A systematic review. Environ. Health Prev. Med..

[37] Moher D., Liberati A., Tetzlaff J., Altman D.G. (2009). The PRISMA Group. Preferred Reporting Items for Systematic Reviews and Meta-Analyses: The PRISMA Statement. PLoS Med..

[38] Pieper D., Antoine S.-L., Mathes T., Neugebauer E.A.M., Eikermann M. (2014). Systematic review finds overlapping reviews were not mentioned in every other overview. J. Clin. Epidemiol..

[39] Yau K.K.-Y., Loke A.Y. (2020). Effects of forest bathing on pre-hypertensive and hypertensive adults: A review of the literature. Environ. Health Prev. Med..

[40] Grilli G., Sacchelli S. (2020). Health Benefits Derived from Forest: A Review. Int. J. Environ. Res. Public Health.

[41] Hansen M.M., Jones R. (2020). The Interrelationship of Shinrin-Yoku and Spirituality: A Scoping Review. J. Altern. Complement. Med..

[42] Rajoo K.S., Karamb D.S., Abdullaha M.Z. (2020). The physiological and psychosocial effects of forest therapy: A systematic review. Urban For. Urban Green..

[43] Kang M.-J., Myung S.-K. (2020). Effects of Forest-Based Interventions on Mental Health; A Meta-Analysis of Randomized Controlled Trials. https://www.crd.york.ac.uk/prospero/display_record.php?ID=CRD42020202489.

[44] Ioannou S., Patterson L., Whittaker P. (2019). The Health Benefits of Forest Therapy: An Umbrella Review. https://www.crd.york.ac.uk/prospero/display_record.php?ID=CRD42019136586.

[45] Kemper H. (2018). Waldbaden: Der Wald Wirkt Entschleunigend.

[46] Markert R. (2019). Alternative Medizin. Waldtherapie—Gesund durch Natur?. https://www.zdf.de/nachrichten/heute/alternative-medizin-heilung-durch-waldtherapie-100.html.

[47] Abe T., Hisama M., Tanimoto S., Shibayama H., Mihara Y., Nomura M. (2008). Antioxidant effects and antimicrobial activites of phytoncide. Biocontrol. Sci..

[48] Li Q., Morimoto K., Kobayashi M., Inagaki H., Katsumata M., Hirata Y., Hirata K., Suzuki H., Li Y.J., Wakayama Y. (2008). Visiting a forest, but not a city, increases human natural killer activity and expression of anti-cancer proteins. Int. J. Immunopathol. Pharmacol..

[49] Šimpraga M., Ghimire R.P., Van Der Straeten D., Blande J.D., Kasurinen A., Sorvari J., Holopainen T., Adriaenssens S., Holopainen J.K., Kivimaenpaa M. (2019). Unravelling the functions of biogenic volatiles in boreal and temperate forest ecosystems. Eur. J. For. Res..

[50] Antonelli M., Donelli D., Barbieri G., Valussi M., Maggini V., Firenzuoli F. (2020). Forest Volatile Organic Compounds and Their Effects on Human Health: A State-of-the-Art Review. Int. J. Environ. Res. Public Health.

[51] Park B.J. Forest Welfare Policy in South Korea. Poster Präsentation am 2. Proceedings of the Internationalen Kongress „Gesundheitspotential Wald“.

[52] Clifford A. (2018). Your Guide to Forest Bathing.

[53] Imai M., Li Q. (2012). An introduction to the Forest Therapy Society of Japan, Forest Therapy and Forest Therapist. Forest Medicine.

[54] Kang B., Kim T., Kim M.J., Lee K.H., Choi S., Lee D.H., Kim H.R., Jun B., Park S.Y., Lee S.J. (2015). Relief of chronic posterior neck pain depending on the type of forest therapy: Comparison of the therapeutic effect of forest bathing alone versus forest bathing with exercise. Ann. Rehabil. Med..

